# One-pot nucleophilic substitution–double click reactions of biazides leading to functionalized bis(1,2,3-triazole) derivatives

**DOI:** 10.3762/bjoc.19.101

**Published:** 2023-09-18

**Authors:** Hans-Ulrich Reissig, Fei Yu

**Affiliations:** 1 Institut für Chemie und Biochemie, Freie Universität Berlin, Takustrasse 3, D-14195 Berlin, Germany; 2 Asymchem Boston Corporation, 10 Gill Street, Woburn, Massachusetts, 01801, USA

**Keywords:** alkynes, azides, copper catalysis, nucleophilic substitution, 1,2-oxazines

## Abstract

The nucleophilic substitution of benzylic bromides with sodium azide was combined with a subsequent copper-catalyzed (3 + 2) cycloaddition with terminal alkynes. This one-pot process was developed with a simple model alkyne, but then applied to more complex alkynes bearing enantiopure 1,2-oxazinyl substituents. Hence, the precursor compounds 1,2-, 1,3- or 1,4-bis(bromomethyl)benzene furnished geometrically differing bis(1,2,3-triazole) derivatives. The use of tris[(1-benzyl-1*H*-1,2,3-triazol-4-yl)methyl]amine (TBTA) as ligand for the click step turned out to be very advantageous. The compounds with 1,2-oxazinyl end groups can potentially serve as precursors of divalent carbohydrate mimetics, but the reductive cleavage of the 1,2-oxazine rings to aminopyran moieties did not proceed cleanly with these compounds.

## Introduction

The concept of click reactions [1–2], in particular, the discovery of the copper-catalyzed alkyne azide (3 + 2) cycloaddition (CuAAC) [3–4], has dramatically changed the approaches to many problems in chemistry, supramolecular chemistry, materials science, biological chemistry and related fields (selected reviews: [5–15]). Mechanistic aspects of the CuAAC have been studied in detail [16–17]. Whereas the traditional 1,3-dipolar cycloaddition (Huisgen reaction) [18–20] of azides and alkynes requires often – but not always – relatively harsh conditions and proceeds with moderate regioselectivity only [21], the copper-catalyzed version can generally be executed at room temperature and it affords exclusively 1,4-disubstituted 1,2,3-triazole derivatives, thus allowing a controlled and highly efficient connection of a variety of molecular building blocks. This “Lego-approach” found countless applications and the bestowal of the Nobel Prize in 2022 to M. Meldal, K. B. Sharpless and C. R. Bertozzi did not come as a surprise.

In most cases the (3 + 2) cycloadditions were performed with isolated (and purified) organic azides, but it was early found that one-pot processes generating the potentially hazardous azides [22] in situ are possible [23]. Later, examples were published showing that these methods are also compatible with the conditions of CuAAC. The earliest case was probably published by Fokin et al. [24–25], one of the inventors of the original copper-catalyzed (3 + 2) cycloaddition. Many examples of nucleophilic substitutions employing sodium azide and organic substrates with potential leaving groups have been reported. The resulting organic azides were trapped in situ by a suitable alkyne to give the 1,2,3-triazoles [26–36]. Fairly recent review articles summarize these results [37–38].

**Scheme 1:**
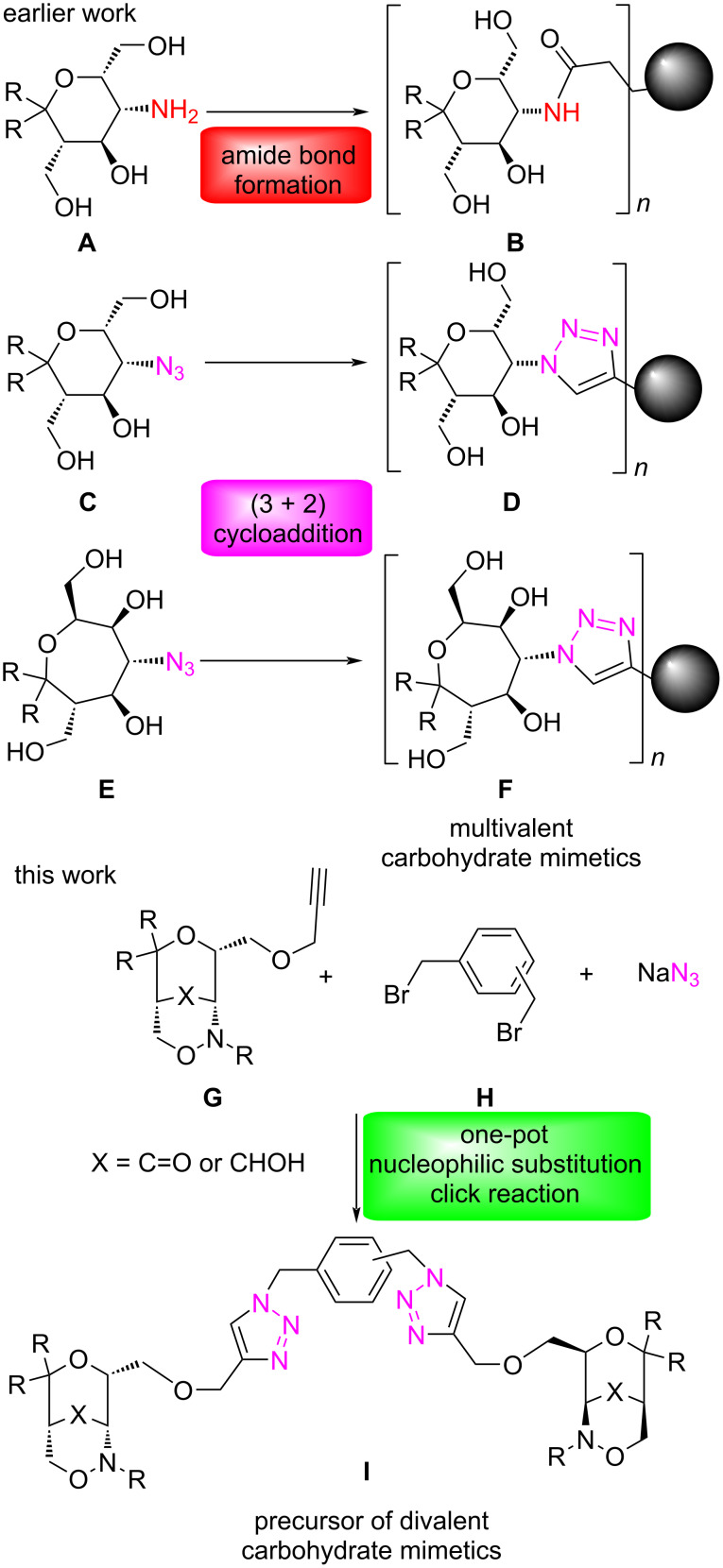
Earlier approaches to multivalent carbohydrate mimetics **B**, **D** or **F** based on enantiopure aminopyran and aminooxepane derivatives and goal of this study employing alkyne component **G** and biazides in situ-generated from **H**.

For several years, our group was interested in preparing multivalent carbohydrate mimetics [39–43] on the basis of efficient coupling reactions of aminopyran and aminooxepane derivatives with suitable linker elements. Hence, the aminopyran derivatives **A** could be converted into several multivalent compounds **B** by amine or amide bond formations [44–47]. The transformation of the corresponding azidopyrans and azidooxepanes **C** or **E** into multivalent 1,2,3-triazole derivatives **D** and **F** by Meldal–Sharpless cycloadditions with suitable alkynes proceeded generally in good yields and furnished another set of multivalent carbohydrate mimetics [48–50]. In the current report, we want to disclose our experience with an “inverted” approach to multivalent systems [51]: bicyclic 1,2-oxazine derivatives of type **G** [52–53], which can be regarded as internally protected aminopyrans [54], should be converted into divalent compounds via coupling of the terminal propynyl group with benzylic biazides. Since biazides are potentially explosive [22] it was very desirable to avoid their isolation and to generate these reactive species in situ from the corresponding benzylic halides. In this study we investigated the compatibility of the nucleophilic substitution of 1,2-, 1,3- or 1,4-bis(bromomethyl)benzene **H** with sodium azide and the copper-catalyzed alkyne–azide cycloadditions with compounds of type **G** to provide divalent compounds **I** (Scheme 1). These may serve as precursors of divalent carbohydrate mimetics with aminopyran end groups.

## Results and Discussion

**Scheme 2:**
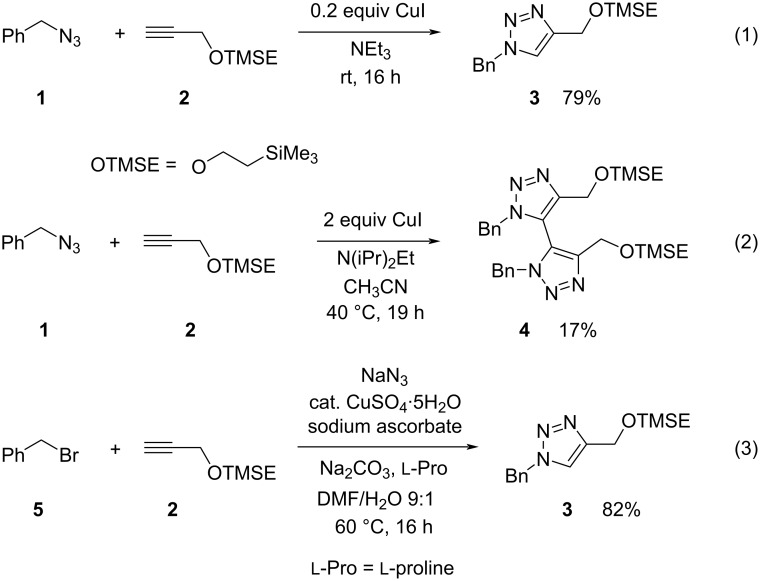
Synthesis of model compound **3** under conventional conditions and as a one-pot process employing benzyl bromide (**5**), alkyne **2** and sodium azide as precursors for the click reaction.

We started our investigations by preparing the [2-(trimethylsilyl)ethoxy]methyl-substituted 1,2,3-triazole derivative **3** as simple model compound applying different conditions (Scheme 2). The highest yield for **3** was recorded when benzyl azide (**1**) and the simple alkyne **2** were combined in the presence of 0.2 equiv of copper(I) iodide in triethylamine as solvent (Scheme 2, reaction 1). After 16 hours at room temperature and chromatographic purification compound **3** was isolated in 79% yield as colorless liquid. Interestingly, under similar conditions, but employing two equivalents of copper(I) iodide in the presence of Hünig’s base [55] in acetonitrile at 40 °C provided 4,4'-bis(1,2,3-triazole) **4** in low yield (Scheme 2, reaction 2). Performing this reaction at room temperature gave a mixture of **3** and **4**. The formation of oxidative dimers in minor amounts has already been observed by Sharpless and co-workers in their original report on CuAAC reactions [4] and was systematically investigated by Burgess et al. [56] who found that the base plays a crucial role during the formation of this type of bistriazoles. This dimerization was also discussed in a review article [57] dealing with the various types of bis(1,2,3-triazoles). Since we were not interested in compounds such as **4** we did not further investigate details in order to optimize this process. Instead, we looked at the one-pot nucleophilic substitution to generate benzyl azide **3** in situ from benzyl bromide (**5**) and sodium azide and to directly trap the intermediate with alkyne **2**. Under conditions summarized in reaction 3 of Scheme 2 we obtained the desired 1,2,3-triazole derivative **3** in 82% yield. Copper(II) sulfate pentahydrate (0.07 equivalents based on **2**) in the presence of sodium ascorbate as reducing agent and sodium carbonate as base as well as ʟ-proline as ligand in a DMF/water mixture at 60 °C provided this promising result. These conditions applied are similar to those described by Fokin et al. [24], which had also been employed by other groups [37–38].

**Scheme 3:**
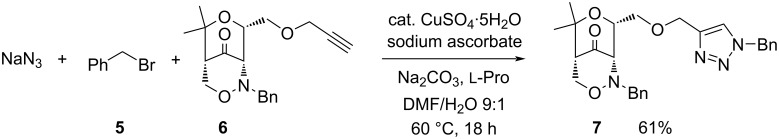
One-pot reaction employing enantiopure alkynyl-substituted 1,2-oxazin-4-one derivative **6** leading to 1,2,3-triazole **7**.

The conditions found were studied with a second model reaction in order to examine its suitability for the synthesis of potential carbohydrate mimetic precursors (Scheme 3). Sodium azide, benzyl bromide (**5**) and enantiopure bicyclic 1,2-oxazin-4-one derivative **6** [53] which bears a propargylic ether moiety as substituent undergo a one-pot reaction with reasonable efficiency and furnished the expected 1,2,3-triazole derivative **7** in 61% yield under the approved conditions.

**Scheme 4:**
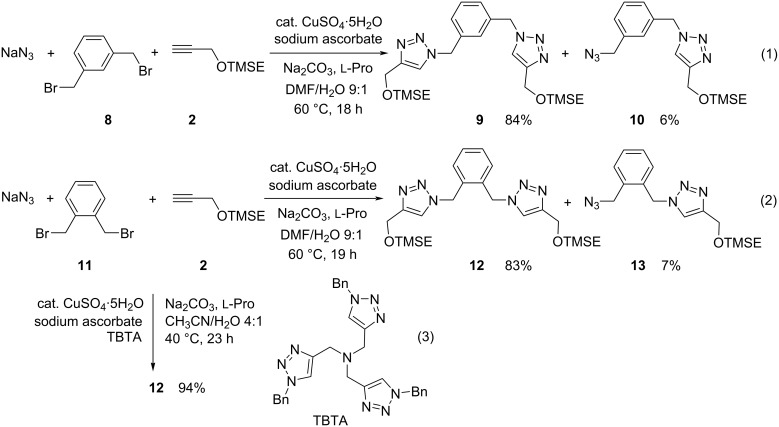
One-pot reactions of dihalides **8** and **11** with sodium azide and alkyne **2** leading to symmetric divalent bis(1,2,3-triazoles) **9** and **12** as major products.

Next, we turned our attention to the generation of divalent systems, starting with the reactions of 1,3- (**8**) and 1,2-bis(bromomethyl)benzene (**11**), respectively, in the presence of sodium azide and alkyne **2** (Scheme 4). The conditions employed above converted the *meta*-substituted dihalide into the expected symmetric bis(1,2,3-triazole) derivative **9** in very good yield, but we also isolated azide **10** in small quantities, where the intermediate biazide has undergone only one cycloaddition (Scheme 4, reaction 1). Under the same conditions the *ortho*-dihalide **11** delivered a very similar result, furnishing the expected product **12** in 83% yield. But again, the corresponding azide **13** was obtained in low yield (Scheme 4, reaction 2). In order to improve the process we examined the influence of the ligand tris[(1-benzyl-1*H*-1,2,3-triazol-4-yl)methyl]amine (TBTA) which has been identified by Sharpless et al. [58] as a very beneficial component in CuAAC reactions. After comprehensive optimization, we found that the addition of 0.2 equiv of this ligand not only allowed to lower the reaction temperature from 60 °C to 40 °C, but it also induced full consumption of the intermediate biazide derived from dihalide **11** (Scheme 4, reaction 3); 0.2 equiv of copper(II) sulfate pentahydrate, 0.4 equiv of sodium ascorbate and 0.4 equiv of ʟ-proline in very little of acetonitrile/water as solvent furnished the exclusively isolated bis(1,2,3-triazole) derivative **12** in excellent 94% yield. These conditions of the one-pot nucleophilic substitution double-click reaction became the standard reaction conditions and were applied in most of the following experiments. When the reaction of **2** with **11** in the presence of sodium azide was performed under similar conditions, but without TBTA (not shown), 58% of **12** and 12% of **13** were isolated, clearly emphasizing the rate enhancing effect of this ligand on the (3 + 2) cycloaddition step.

**Scheme 5:**
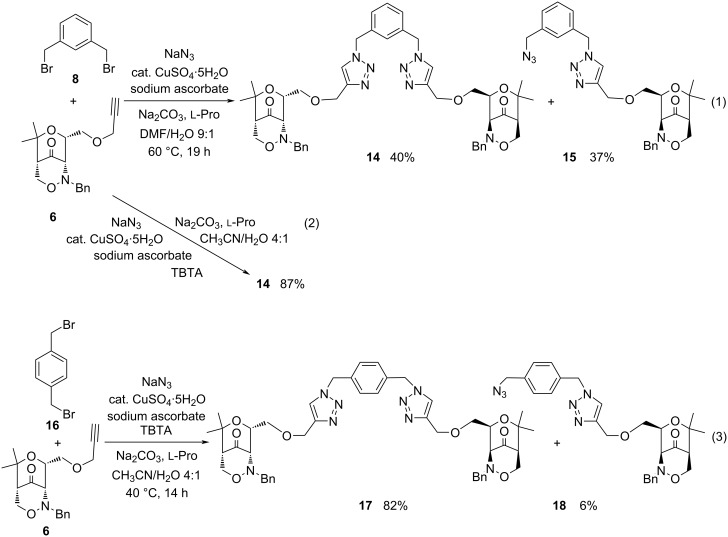
One-pot reactions employing enantiopure alkynyl-substituted 1,2-oxazin-4-one derivative **6** leading to bis(1,2,3-triazoles) **14** and **17** as major products.

With enantiopure alkynyl-substituted 1,2-oxazin-4-one derivative **6**, a potential aminopyran precursor, as alkyne component similar experience was gathered (Scheme 5). This sterically more demanding component reacted slower under the initially examined conditions and provided the desired bis(1,2,3-triazole) **14** and the mono-cycloadduct **15** in almost equal quantities (Scheme 5, reaction 1). Again, the addition of TBTA as ligand strongly improved the performance of the CuAAC reaction: the symmetric divalent compound **14** with two 1,2-oxazinyl end groups was isolated in very satisfying 87% yield (Scheme 5, reaction 2). When 1,4-bis(bromomethyl)benzene (**16**) was employed as starting material, these conditions afforded the *para*-substituted bis(1,2,3-triazole) **17** in very good yield although the azidomethyl-substituted compound **18** was also isolated in 6% yield in this case (Scheme 5, reaction 3).

**Scheme 6:**
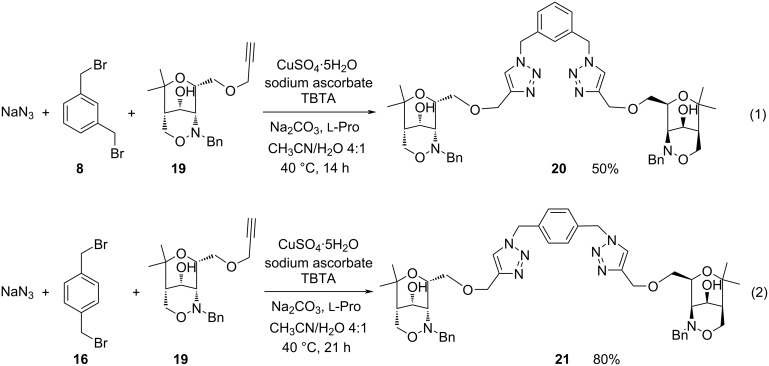
One-pot reaction employing enantiopure alkynyl-substituted 1,2-oxazin-4-ol derivative **19** leading to bis(1,2,3-triazoles) **20** and **21**.

Finally, the one-pot click reactions were examined with the alkynyl-substituted 1,2-oxazin-4-ol derivative **19** which is smoothly available from the corresponding ketone **6** by highly stereoselective reduction with sodium borohydride [53]. Starting from 1,3- (**8**) or 1,4-bis(bromomethyl)benzene (**16**), respectively, the approved conditions in the presence of TBTA led to the expected bis(1,2,3-triazoles) **20** or **21** in moderate or very good yield (Scheme 6). We cannot decide whether the lower yields in this series are caused by the unprotected hydroxy group of precursor **19** or the corresponding products. Although we did not isolate the conceivable mono-adducts we cannot rigorously exclude their formation.

**Scheme 7:**
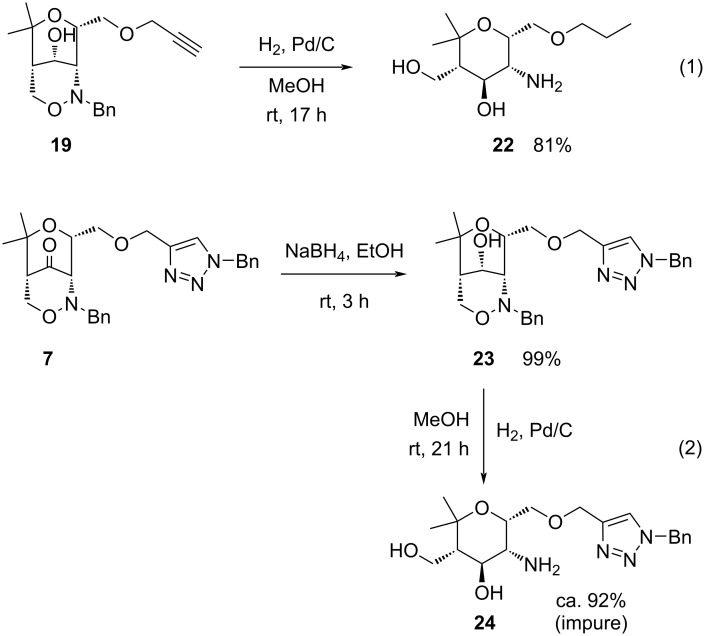
Reductive ring-openings of 1,2-oxazine derivatives **19** and **23** as simple model compounds by hydrogenolysis in the presence of palladium on carbon leading to aminopyran derivatives **22** and **24**.

Compared to bis(1,2,3-triazoles) **14** and **17**, compounds **20** and **21** are one step closer to the desired divalent aminopyran-substituted carbohydrate mimetics, since they already contain a free hydroxy group instead of the carbonyl group. However, their reductive transformation into divalent carbohydrate mimetics turned out to be a difficult task. We started the experiments with the hydrogenolysis of bicyclic 1,2-oxazin-4-ol **19** as simple model compound (Scheme 7). A methanol solution of **19** under an atmosphere of hydrogen was stirred for 17 h in the presence of palladium on carbon and provided the expected (propyloxy)methyl-substituted aminopyran **22** in 81% yield (Scheme 7, reaction 1). The reductive removal of the *N*-benzyl group and the cleavage of the N–O bond occurred apparently without problems. With the second model compound, 1,2,3-triazole **23**, which is almost quantitatively available by sodium borohydride reduction of **7**, we encountered the first problems. Under similar conditions of the hydrogenolysis the expected product **24** could be isolated as major component and characterized (Scheme 7, reaction 2), but the obtained sample contained unknown byproducts. It is possible, that the *N*-benzyl group attached to the 1,2,3-triazole moiety is partially removed under these conditions and/or that even the C–O bond connecting the 1,2,3-triazole part with the aminopyran part is reductively cleaved since this bond also has benzylic character. In earlier investigations with other triazolyl-substituted aminopyran derivatives we encountered similar selectivity problems due to the sensitivity of benzylic bonds to the applied hydrogenolysis conditions [59].

**Scheme 8:**
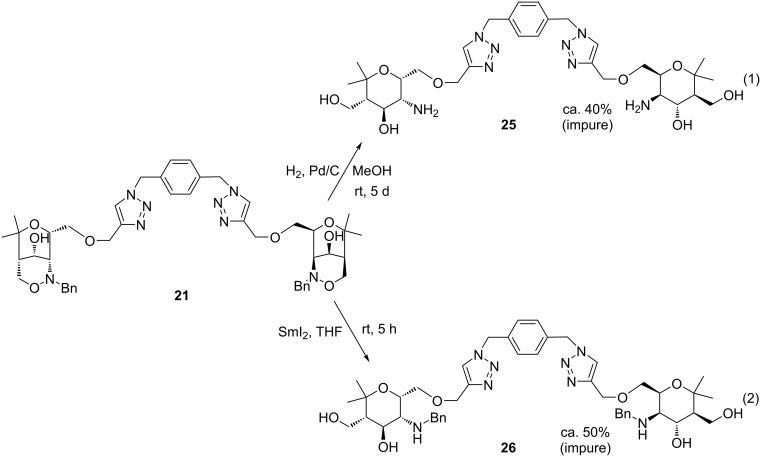
Attempted reductive ring-openings of compound **21** by hydrogenolysis or by samarium diiodide leading to impure samples of divalent aminopyran derivatives **25** or **26**.

Despite of the discouraging results with model compound **23** we nevertheless examined the reductive cleavage of bis(1,2,3-triazole) **21**. It turned out that the hydrogenolysis of this compound was very capricious and (in part) depended on the batch of palladium on carbon used. In most cases, incomplete consumption of starting material was observed, even after long reaction time. The best result is depicted in Scheme 8 (reaction 1), when after five days of hydrogenolysis and 0.8 equivalents of palladium at least 40% of impure divalent aminopyran derivative **25** was isolated. The major component of the isolated material is certainly fitting to the proposed structure according to the NMR data and their comparison with related compounds, but the sample again contains unknown impurities, which are probably due to additional bond cleavage events. Compound **25** contains four bonds of heteroatoms to carbon atoms which have benzylic character and are possibly attacked under the reaction conditions, in particular, considering the long reaction time and the fairly high amount of catalyst employed.

As an alternative method, which should be more chemoselective, we examined the reduction with samarium diiodide [60]. This versatile one-electron transfer reagent is known to cleave N–O bonds with high selectivity [61–62] and was applied several times by our group with good success for reductive ring-openings of 1,2-oxazine derivatives [63–64]. A slight excess of samarium diiodide in tetrahydrofuran completely consumed compound **21** within five hours. Compound **26** was isolated in 50% yield, but again the sample was not clean. The NMR data revealed that the *N*-benzyl groups were still intact, however, we cannot exclude that partial reductions had occurred at other positions of this relatively complex molecule. Thus, better conditions for the clean transformation of compounds such as **21** into carbohydrate mimetics have still to be developed. We abstained from the conversion of compounds **24**, **25** or **26** into their sulfated form due to the presence of the impurities in these samples. However, only the sulfated forms of this type of multivalent carbohydrate mimetics had earlier shown respectable biological activity as ligands of P- and L-selectins [44–50].

## Conclusion

We found very good conditions for a one-pot nucleophilic substitution of benzylic bromides with sodium azide and direct subsequent CuAAC reactions with alkynes. The conditions were tested with the simple alkyne model compound **2**, but could be applied to more complex alkynes such as 1,2-oxazin-4-one derivative **6** or 1,2-oxazin-4-ol derivative **19**. With 1,2-, 1,3- or 1,4-bis(bromomethyl)benzene as precursors bis(1,2,3-triazole) derivatives were isolated in good to excellent yields. The use of TBTA as ligand for the click step turned out to be very advantageous. The reductive ring openings of bis(1,2,3-triazole) derivative **21** to divalent carbohydrate mimetics with hydrogen under palladium catalysis or with samarium diiodide did not proceed cleanly and need further optimization.

## Supporting Information

File 1 Experimental procedures, spectroscopic and analytical characterization data of new compounds as well as copies of the NMR spectra.
