# Supplementary material for: One-pot nucleophilic substitution–double click reactions of biazides leading to functionalized bis(1,2,3-triazole) derivatives
Source: Beilstein J Org Chem. 2023 Sep 18;19:1399–407. doi: 10.3762/bjoc.19.101 (PMC10520474; doi:10.3762/bjoc.19.101)
Supplement: File 1 — Experimental procedures, spectroscopic and analytical characterization data of new compounds as well as copies of the NMR spectra. [file Beilstein_J_Org_Chem-19-1399-s001.pdf]

BEILSTEIN JOURNAL OF ORGANIC CHEMISTRY

Supporting Information

for

One-pot nucleophilic substitution–double click reactions of biazides leading to functionalized bis(1,2,3-triazole) derivatives

Hans-Ulrich Reissig and Fei Yu

Beilstein J. Org. Chem. **2023,** *19,* 1399–1407. doi:10.3762/bjoc.19.101

Experimental procedures, spectroscopic and analytical characterization data of new compounds as well as copies of the NMR spectra

License and Terms: This is a supporting information file under the terms of the Creative Commons Attribution License (https://creativecommons.org/licenses/by/4.0). Please note that the reuse, redistribution and reproduction in particular requires that the author(s) and source are credited and that individual graphics may be subject to special legal provisions.
The license is subject to the *Beilstein Journal of Organic Chemistry* terms and conditions: (https://www.beilstein-journals.org/bjoc/terms)

Tables of contents

1. General information S2

2. Synthesis and characterization of compounds S3

3. References S13

4. Copies of ^{1}H NMR and ^{13}C NMR spectra S14

1. General information

Reactions were performed under argon in flame-dried flasks, if not stated otherwise. Liquid components were added by syringe. Tetrahydrofuran and dichloromethane were obtained from a solvent purification system MB-SPS-800 (M. Braun). Methanol was purchased in p. a. quality and stored under argon over molecular sieves (4 Å). Products were purified by flash chromatography on aluminum oxide. Unless otherwise stated, yields refer to analytically pure samples. ^{1}H NMR [$CHCl_3$ (δ = 7.26 ppm), TMS (δ = 0.00 ppm), or CD_3OD (δ = 3.31 ppm) as internal standards] and ^{13}C NMR spectra [$CDCl_3$ (δ = 77.0 ppm), or CD_3OD (δ = 49.0 ppm) as internal standards] were recorded on Bruker AC 500, or Joel Eclipse 500 instruments in $CDCl_3$ or CD_3OD solution. Integrals are in accordance with assignments; coupling constants are given in Hz. IR spectra were measured with an FT-IR spectrometer Nicolet 5 SXC or with a Nexus FT-IR equipped with a Nicolet Smart Dura Sample IR ATR. HRMS analyses were performed on an Agilent ESI-TOF 6210 (4 µL/min, 1 bar, 4000 V) instrument. The elemental analyses were recorded with "Elemental-Analyzers" (Perkin–Elmer or Carlo Erba). Melting points were measured with a Reichert apparatus (Thermovar) and are uncorrected. Optical rotations ($[\alpha]_D$) were determined with Perkin–Elmer 241 polarimeter at the temperatures given. Commercially available chemicals were used without further purification unless otherwise stated.

2. Synthesis and characterization of compounds

1-Benzyl-4-{[2-(trimethylsilyl)ethoxy]methyl}-1*H*-1,2,3-triazole (3): A mixture of benzyl azide (**1**) [1] (67 mg, 0.50 mmol), alkyne **2** [2] (78 mg, 1.00 mmol), CuI (19 mg, 0.10 mmol), and triethylamine (1.45 g, 1.43 mmol) was under air atmosphere at room temperature for 16 h. Ethyl acetate (20 mL) was added, the reaction mixture was washed with aqueous ammonia solution (25%, 2 × 10 mL) and dried (Na_2SO_4). After filtration and evaporation, the residue was purified by column chromatography (aluminum oxide, hexanes/ethyl acetate, 3:1) to provide **3** (114 mg, 79%) as colorless liquid.

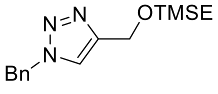

3

^{1}H NMR ($CDCl_3$, 500 MHz): δ = –0.07 (s, 9 H), 0.89 (m_c, 2 H), 3.53 (m_c, 2 H), 4.53 (s, 2 H), 5.44 (s, 2 H), 7.17–7.20, 7.22–7.32 (2 m, 2 H, 3 H), 7.41 (s, 1 H); ^{13}C NMR ($CDCl_3$, 125 MHz): δ = –1.6, 18.0, 53.8, 63.6, 67.7, 122.1, 127.8, 128.4, 128.8, 134.4, 145.9; IR (KBr): υ = 3065, 3035, 2950, 2860, 1495, 1455 cm^{-1}; HRMS (ESI-TOF): *m/z* $[M + Na]^+$ calcd for $C_{15}H_{23}N_3NaOSi$: 312.1508; found: 312.1485; Anal. calcd for $C_{15}H_{23}N_3OSi$ (289.5): C, 62.24; H, 8.01; N, 14.52; found: C, 61.96; H, 8.05; N, 14.55.

3,3´-Dibenzyl-5,5´-bis-{[2-(trimethylsilyl)ethoxy]methyl}-3*H*,3´*H*-4,4´-bi(1,2,3-triazole) (4): A mixture of benzyl azide (**1**) (196 mg, 1.47 mmol), **2** (160 mg, 1.02 mmol), CuI (383 mg, 2.01 mmol), and $N(iPr)_2Et$ (370 mg, 2.86 mmol) in acetonitrile (2 mL) was stirred at 40 °C for 19 h. Ethyl acetate (50 mL) was added, the reaction mixture was twice washed with aqueous ammonia solution (25%, 2 × 10 mL) and dried (Na_2SO_4). After filtration and evaporation, the residue was purified by column chromatography (aluminum oxide, hexanes/ethyl acetate, 3:1) to give **4** (49 mg, 17%) as colorless liquid.

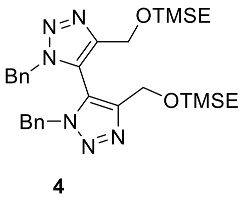

4

^{1}H NMR ($CDCl_3$, 500 MHz): δ = –0.06 (s, 18 H), 0.70–0.79 (m, 4 H), 3.29–3.57 (m, 4 H), 3.91, 4.08 (2 d, *J* = 12.0 Hz, 2 H each), 4.60, 4.98 (2 d, *J* = 15.0 Hz, 2 H each), 6.90 (d, *J* = 6.5 Hz, 4 H), 7.20–7.28 (m, 6 H); ^{13}C NMR ($CDCl_3$, 125 MHz): δ = –1.5, 18.0, 52.3, 62.5, 68.1, 122.1, 128.2, 128.7, 128.8, 134.5, 147.0; IR (ATR): υ = 3065, 3035, 2950, 1495, 1455 cm^{-1}; HRMS (ESI-TOF): calcd for *m/z* $[M + H]^+$ $C_{30}H_{45}N_6O_2Si_2$: 577.3143; found: 577.3138; calcd for *m/z* $[M + Na]^+$

$C_{30}H_{44}N_6NaO_2Si_2$: 599.2962; found: 599.2957; calcd for *m/z* $[M + K]^+$ $C_{30}H_{44}N_6KO_2Si_2$: 615.2701; found: 615.2693; Anal. calcd for $C_{30}H_{44}N_6O_2Si_2$ (576.9): C, 62.46; H, 7.69; N, 14.57; found: C, 61.85; H, 7.50; N, 13.98.

One-pot synthesis of 1-benzyl-4-{[2-(trimethylsilyl)ethoxy]methyl}-1*H*-1,2,3-triazole (3): A mixture of sodium azide (41 mg, 0.63 mmol), benzyl bromide (**5**) (89 mg, 0.52 mmol), alkyne **2** (76 mg, 0.57 mmol), $CuSO_4{\cdot}5H_2O$ (13 mg, 0.052 mmol), sodium ascorbate (21 mg, 0.11 mmol), L-proline (12 mg, 0.10 mmol), and Na_2CO_3 (12 mg, 0.11 mmol) in DMF/H_2O (9:1, 1 mL) was stirred at 60 °C for 16 h. Ethyl acetate (50 mL) was added, the reaction mixture was washed with aqueous ammonia solution (25%, 2 × 10 mL) and dried (Na_2SO_4). After filtration and evaporation, the residue was purified by column chromatography (aluminum oxide, hexanes/ethyl acetate, 3:1) to give **3** (124 mg, 82%) as colorless liquid.

(1*S*,5*R*,8*S*)-2-Benzyl-8-{[(1-benzyl-1*H*-1,2,3-triazol-4-yl)methoxy]methyl}-6,6-dimethyl-3,7-dioxa-2-azabicyclo[3.3.1]nonan-9-one (7): A mixture of benzyl bromide (**5**) (18 mg, 0.11 mmol), alkyne **6** [3] (35 mg, 0.11 mmol), sodium azide (8 mg, 0.12 mmol), $CuSO_4{\cdot}5H_2O$ (2 mg, 0.008 mmol), sodium ascorbate (4 mg, 0.02 mmol), L-proline (3 mg, 0.026 mmol), and Na_2CO_3 (2 mg, 0.019 mmol) was stirred at 60 °C in DMF/H_2O (9:1, 1.0 mL) for 18 h. Ethyl acetate (50 mL) was added, the reaction mixture was washed with aqueous ammonia solution (25%, 2 × 10 mL) and dried (Na_2SO_4). After filtration and evaporation, the residue was purified by column chromatography (aluminum oxide, hexanes/ethyl acetate, 3:1) to give **7** (31 mg, 61%) as colorless liquid.

$[\alpha]_D^{22}$ = 57.2 (*c* = 0.7, MeOH); ^1H NMR ($CDCl_3$, 500 MHz): δ = 1.16, 1.37 (2 s, 3 H each), 2.29 (s, 1 H), 3.17 (s, 1 H), 3.70–3.77, 3.83–3.90 (2 m, 1 H, 2 H), 4.02–4.07 (m, 1 H), 4.10 (d, *J* = 13.5 Hz, 1 H), 4.42–4.50 (m, 2 H), 4.54, 4.61 (AB system, J_{AB} = 12.0 Hz, 2 H), 5.41 (s, 2 H), 7.17 (t, *J* = 7.0 Hz, 1 H), 7.20–7.30, 7.32–7.38 (m, 7 H, 3 H); ^{13}C NMR ($CDCl_3$, 125 MHz): δ = 23.7, 26.7, 54.0, 57.9, 59.6, 64.9, 69.1, 69.2, 69.7, 74.0, 78.3, 122.2, 127.4, 128.0, 128.3, 128.68, 128.73, 129.0, 134.4, 136.1, 145.2; IR (ATR): υ = 3065, 3030, 2930, 2870, 1725, 1495, 1455; HRMS (ESI-TOF): calcd for *m/z* $[M + H]^+$ $C_{26}H_{31}N_4O_4$: 463.2345; found: 463.2362; calcd for *m/z* $[M + Na]^+$ $C_{26}H_{30}N_4NaO_4$: 485.2165; found: 485.2185; calcd for *m/z* $[M + K]^+$ $C_{26}H_{30}N_4KO_4$: 501.1904; found: 501.1926.

1,3-Bis({4-[((2-trimethylsilyl)ethoxy)methyl]-1*H*-1,2,3-triazol-1-yl}methyl)benzene (9) and 1-[3-(azidomethyl)benzyl]-4-{[2-(trimethylsilyl)ethoxy]methyl}-1*H*-1,2,3-triazole (10): A mixture of 1,3-bis(bromomethyl)benzene (**8**) (132 mg, 0.50 mmol), alkyne **2** (189 mg, 1.21

mmol), sodium azide (79 mg, 1.22 mmol), $CuSO_4 \cdot 5H_2O$ (25 mg, 0.10 mmol), sodium ascorbate (40 mg, 0.20 mmol), L-proline (23 mg, 0.20 mmol), and Na_2CO_3 (21 mg, 0.20 mmol) in DMF/H_2O (9:1, 0.5 mL) was stirred at 60 °C for 18 h. Ethyl acetate (50 mL) was added, the reaction mixture was washed with aqueous ammonia solution (25%, 2 × 10 mL) and dried (Na_2SO_4). After filtration and evaporation, the residue was purified by column chromatography (aluminum oxide, hexanes/ethyl acetate, 3:1) to give **9** (209 mg, 84%) and **10** (10 mg, 6%) as colorless liquids.

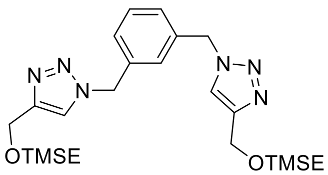

9

^1H NMR ($CDCl_3$, 500 MHz): δ = –0.12 (s, 18 H), 0.84 (m_c, 4 H), 3.49 (m_c, 4 H), 4.46 (s, 4 H), 5.38 (s, 4 H), 7.10 (d, *J* = 8.0 Hz, 2 H), 7.11 (s, 1 H), 7.22 (t, *J* = 8.0 Hz, 1 H), 7.42 (s, 2 H, 5-H); NMR ($CDCl_3$, 125 MHz): δ = –1.7, 17.9, 53.8, 63.4, 67.7, 122.1, 127.3, 127.9, 129.5, 135.5, 145.6; IR (ATR): υ = 3135, 2950, 2860, 1450, 1435 cm^{-1}; HRMS (ESI-TOF): calcd for *m/z* $[M + H]^+$ $C_{24}H_{41}N_6O_2Si_2$: 501.2830; found: 501.2827; calcd for *m/z* $[M + Na]^+$ $C_{24}H_{40}N_6NaO_2Si_2$: 523.2649; found: 523.2648; calcd for *m/z* $[M + K]^+$ $C_{24}H_{40}N_6KO_2Si_2$: 539.2388; found: 539.2384.

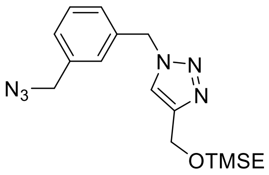

10

^1H NMR ($CDCl_3$, 500 MHz): δ = –0.03 (s, 9 H), 0.93 (m_c, 2 H), 3.57 (m_c, 2 H), 4.33 (s, 2 H), 4.57 (s, 2 H), 5.52 (s, 2 H), 7.19–7.25 (m, 2 H), 7.29 (d, *J* = 8.0 Hz, 1 H), 7.38 (t, *J* = 8.0 Hz, 1 H), 7.45 (s, 1 H); ^{13}C NMR ($CDCl_3$, 125 MHz): δ = –1.5, 18.2, 53.8, 54.3, 63.8, 68.0, 122.2, 127.6, 127.9, 128.4, 129.6, 135.3, 136.5, 146.1; IR (ATR): υ = 3135, 2950, 2855, 2095, 1450 cm^{-1}; HRMS (ESI-TOF): calcd for *m/z* $[M + H]^+$ $C_{16}H_{25}N_6OSi$: 345.1859; found: 345.1862; calcd for *m/z* $[M + Na]^+$ $C_{16}H_{24}N_6NaOSi$: 367.1679; found: 367.1679; calcd for *m/z* $[M + K]^+$ $C_{16}H_{24}N_6KOSi$: 383.1418; found: 383.1419; Anal. calcd for $C_{16}H_{24}N_6OSi$ (344.5): C, 55.78; H, 7.02; N, 24.40; found: C, 56.30; H, 7.12; N, 22.06.

1,2-Bis({4-[((2-trimethylsilyl)ethoxy)methyl]-1*H*-1,2,3-triazol-1-yl}methyl)benzene (12) and 1-[2-(azidomethyl)benzyl]-4-{[2-(trimethylsilyl)ethoxy]methyl}-1*H*-1,2,3-triazole (13): A mixture of 1,2-bis(bromomethyl)benzene (**11**) (134 mg, 0.51 mmol), alkyne **2** (187 mg, 1.20

mmol), sodium azide (78 mg, 1.20 mmol), $CuSO_4 \cdot 5H_2O$ (25 mg, 0.10 mmol), sodium ascorbate (40 mg, 0.20 mmol), L-proline (24 mg, 0.21 mmol), and Na_2CO_3 (23 mg, 0.22 mmol) in DMF/H_2O (9:1, 1.5 mL) was stirred at 60 °C for 19 h. Ethyl acetate (50 mL) was added, the mixture was washed with ammonia solution (25%, 2 × 10 mL) and dried (Na_2SO_4). After filtration and evaporation, the residue was purification by column chromatography (aluminum oxide, hexanes/ethyl acetate, 3:1) to give **12** (213 mg, 83%) as colorless solid (m.p. 71-72 °C) and **13** (12 mg, 7%) as colorless liquid.

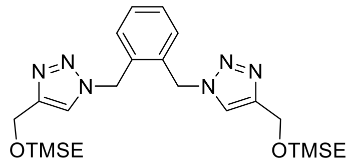

12

^1H NMR ($CDCl_3$, 500 MHz): δ = –0.10 (s, 18 H), 0.86 (m_c, 4 H), 3.50 (m_c, 4 H), 4.47 (s, 4 H), 5.52 (s, 4 H), 7.09–7.14, 7.21-7.27 (2 m, 2 H each), 7.40 (s, 2 H); ^{13}C NMR ($CDCl_3$, 125 MHz): δ = –1.6, 17.9, 50.8, 63.4, 67.8, 122.4, 129.4, 130.0, 133.1, 145.7; IR (KBr): υ = 3135, 3040, 2950, 2865, 1460 cm^{-1}; HRMS (ESI-TOF): calcd for *m/z* $[M + H]^+$ $C_{24}H_{41}N_6O_2Si_2$: 501.2830; found: 501.2800; calcd for *m/z* $[M + Na]^+$ $C_{24}H_{40}N_6NaO_2Si_2N$: 523.2649; found: 523.2620; calcd for *m/z* $[M + K]^+$ $C_{24}H_{40}N_6KO_2Si_2$: 539.2388; found: 539.2357; Anal. calcd for $C_{24}H_{40}N_6O_2Si_2$ (500.8): C, 57.56; H, 8.05; N, 16.78; found: C, 57.26; H, 8.09; N, 16.69.

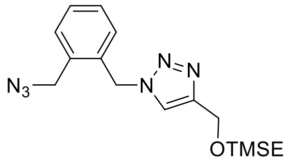

13

^1H NMR ($CDCl_3$, 500 MHz): δ = –0.02 (s, 9 H), 0.94 (m_c, 2 H), 3.58 (m_c, 2 H), 4.42 (s, 2 H), 4.58 (s, 2 H), 5.60 (s, 2 H), 7.23 (d, *J* = 7.5 Hz, 1 H), 7.32–7.40 (m, 3 H), 7.44 (s, 1 H); ^{13}C NMR ($CDCl_3$, 125 MHz): δ = –1.5, 18.1, 51.1, 52.5, 63.8, 68.0, 122.2, 129.3, 129.4, 130.0, 130.3, 133.2, 133.8, 146.0; IR (ATR): υ = 3135, 3070, 2950, 2860, 2095, 1455 cm^{-1}; HRMS (ESI-TOF): calcd for *m/z* $[M + H]^+$ $C_{16}H_{25}N_6OSi$: 345.1859; found: 345.1857; calcd for *m/z* $[M + Na]^+$ $C_{16}H_{24}N_6NaOSi$: 367.1679; found: 367.1677; calcd for *m/z* $[M + K]^+$ $C_{16}H_{24}N_6KOSi$: 383.1418; found: 383.1414; Anal. calcd for $C_{16}H_{24}N_6OSi$ (344.5): C, 55.78; H, 7.02; N, 24.40; found: C, 56.35; H, 7.15; N, 23.12.

Representative procedure (RP) for the synthesis of divalent compounds by click-reactions in the presence of TBTA, synthesis of bis(1,2,3-triazole) 12: A mixture of 1,2-

bis(bromomethyl)benzene (**11**) (67 mg, 0.25 mmol), alkyne **2** (86 mg, 0.55 mmol), sodium azide (40 mg, 0.62 mmol), $CuSO_4{\cdot}5H_2O$ (13 mg, 0.05 mmol), sodium ascorbate (20 mg, 0.10 mmol), tris[(1-benzyl-1*H*-1,2,3-triazol-4-yl]methyl)amine (TBTA) [4] (27 mg, 0.05 mmol), L-proline (12 mg, 0.10 mmol), and Na_2CO_3 (11 mg, 0.10 mmol) in CH_3CN/H_2O (4:1, 0.5 mL) was stirred at 40 °C for 23 h. Ethyl acetate (50 mL) was added, the reaction mixture was washed with aqueous ammonia solution (25%, 2 × 10 mL) and dried (Na_2SO_4). After filtration and evaporation, the residue was purified by column chromatography (aluminum oxide, hexanes/ethyl acetate, 3:1) to give **12** (117 mg, 94%) as colorless solid.

1,3-Bis(1,2,3-triazole) 14: Following the **RP**, a mixture of 1,3-bis(bromomethyl)benzene (**8**) (8 mg, 0.030 mmol), alkyne **6** (24 mg, 0.073 mmol), sodium azide (5 mg, 0.077 mmol), $CuSO_4{\cdot}5H_2O$ (2 mg, 0.008 mmol), sodium ascorbate (2 mg, 0.010 mmol), TBTA (3 mg, 0.006 mmol), L-proline (2 mg, 0.017 mmol), and Na_2CO_3 (1 mg, 0.010 mmol) in CH_3CN/H_2O (4:1, 0.3 mL) was stirred at 40 °C for 18 h. Standard work-up and purification by column chromatography (aluminum oxide hexanes/ethyl acetate, 3:1) gave **14** (22 mg, 87%) as colorless liquid.

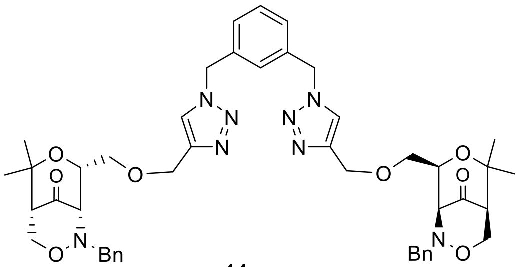

14

$[\alpha]_D^{22}$ = 54.4 (*c* = 0.7, MeOH); ^{1}H NMR ($CDCl_3$, 500 MHz): δ = 1.17, 1.38 (2 s, 6 H), 2.29 (s, 2 H), 3.17 (s, 2 H), 3.71–3.78 (m, 2 H), 3.83–3.91 (m, 4 H), 4.06 (t, *J* = 7.0 Hz, 2 H), 4.11 (d, *J* = 14.0 Hz, 2 H), 4.44–4.50 (m, 4 H), 4.55, 4.63 (AB system, J_{AB} = 12.0 Hz, 4 H), 5.36, 5.40 (AB system, J_{AB} = 15.0 Hz, 4 H), 7.11 (s, 2 H), 7.14–7.20, 7.22–7.30 (2 m, 4 H, 9 H), 7.33 (t, *J* = 7.5 Hz, 1 H); ^{13}C NMR ($CDCl_3$, 125 MHz): δ = 23.8, 26.7, 53.5, 58.0, 59.6, 64.8, 69.1, 69.4, 69.8, 74.1, 78.3, 122.4, 127.4, 127.5, 128.2, 128.3, 128.8, 129.9, 135.7, 136.1, 145.4, 208.2; IR (ATR): *υ* = 2925, 2870, 1725, 1495, 1455 cm^{-1}; HRMS (ESI-TOF): calcd for *m/z* $[M + H]^+$ $C_{46}H_{55}N_8O_8$: 847.4143; found: 847.3999; calcd for *m/z* $[M + Na]^+$ $C_{46}H_{54}N_8NaO_8$: 869.3963; found: 869.3920; calcd for *m/z* $[M + Na]^+$ $C_{46}H_{54}N_8KO_8$: 885.3702; found: 885.3816.

1,3-Bis(1,2,3-triazole) 14 and azidomethyl(1,2,3)-triazole 15: A mixture of 1,3-bis(bromomethyl)benzene (**8**) (26 mg, 0.099 mmol), alkyne **6** (80 mg, 0.24 mmol), sodium azide (16 mg, 0.25 mmol), $CuSO_4{\cdot}5H_2O$ (5 mg, 0.020 mmol), sodium ascorbate (8 mg, 0.040

mmol), L-proline (5 mg, 0.043 mmol), and Na_2CO_3 (4 mg, 0.038 mmol) in DMF/H_2O (9:1, 0.5 mL) was stirred at 60 °C 17 h. Ethyl acetate (50 mL) was added, the mixture was washed with aqueous ammonia solution (25%, 2 × 10 mL) and dried (Na_2SO_4). After filtration and evaporation, the residue was purified by column chromatography (aluminum oxide, hexanes/ethyl acetate, 3:1) to give **14** (34 mg, 40%) and **15** (19 mg, 37%) as colorless liquids. Data of **14**, see above.

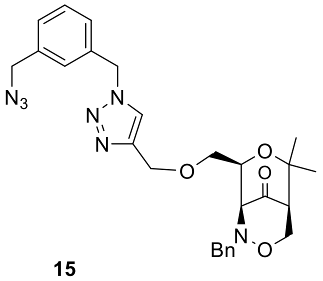

15

$[\alpha]_D^{22}$ = 19.3 (*c* = 0.9, MeOH); ^{1}H NMR ($CDCl_3$, 500 MHz): δ = 1.17, 1.38 (2 s, 3 H each), 2.29 (s_{br}, 1 H), 3.18 (s_{br}, 1 H), 3.72–3.78, 3.84–3.91 (2 m, 1 H, 2 H), 4.05 (dt, *J* = 2.0, 7.0 Hz, 1 H), 4.11 (d, *J* = 13.5 Hz, 1 H), 4.32 (s, 2 H), 4.42–4.52 (m, 2 H), 4.55, 4.63 (2 d, *J* = 12.0 Hz, 1 H each), 5.41, 5.45 (AB system, J_{AB} = 15.0 Hz, 2 H), 7.15–7.21, 7.22–7.38 (2 m, 3 H, 6 H), 7.38 (t, *J* = 8.0 Hz, 1 H); ^{13}C NMR ($CDCl_3$, 125 MHz): δ = 23.8, 26.7, 53.8, 54.3, 58.0, 59.6, 64.9, 69.1, 69.4, 69.9, 74.1, 78.3, 122.2, 127.5, 127.6, 127.9, 128.4, 128.8, 129.7, 135.3, 136.2, 136.5, 145.4, 208.2; IR (ATR): *υ* = 3065, 3030, 2925, 2870, 2095, 1725, 1495, 1455 cm^{-1}; HRMS (ESI-TOF): calcd for *m/z* $[M + Na]^+$ $C_{27}H_{31}N_7NaO_4$: 540.2335; found: 540.2294.

1,4-Bis(1,2,3-triazole) 17 and azidomethyl(1,2,3-triazole) 18: Following the **RP**, a mixture of 1,4-bis(bromomethyl)benzene (**16**) (26 mg, 0.099 mmol), alkyne **6** (80 mg, 0.24 mmol), sodium azide (17 mg, 0.26 mmol), $CuSO_4{\cdot}5H_2O$ (5 mg, 0.02 mmol), sodium ascorbate (8 mg, 0.04 mmol), TBTA (11 mg, 0.021 mmol), L-proline (5 mg, 0.043 mmol), and Na_2CO_3 (4 mg, 0.038 mmol) in CH_3CN/H_2O (4:1, 0.5 mL) was stirred at 40 °C for 14 h. Standard work-up and purification by column chromatography (aluminum oxide, hexanes/ethyl acetate, 3:1) gave **17** (70 mg, 82%) and **18** (3 mg, 6%) as colorless liquids.

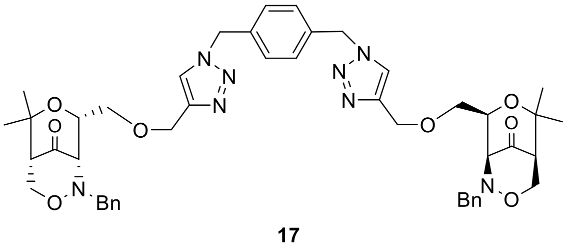

17

$[\alpha]_D^{22}$ = 52.8 (*c* = 0.8, MeOH); ^{1}H NMR ($CDCl_3$, 500 MHz): δ = 1.15, 1.36 (2 s, 6 H each), 2.28 (t,

J = 2.0 Hz, 2 H), 3.15 (s, 2 H), 3.70–3.76, 3.83–3.90 (2 m, 2 H, 4 H), 4.01–4.05 (m, 2 H), 4.09 (d, J = 14.0 Hz, 2 H), 4.44–4.49 (m, 4 H), 4.52, 4.60 (2 d, J = 12.0 Hz, 2 H each), 5.36–5.40 (m, 4 H), 7.14–7.19, 7.20–7.30 (2 m, 6 H, 10 H); ^{13}C NMR ($CDCl_3$, 125 MHz): δ = 23.7, 26.6, 53.4, 57.9, 59.6, 64.8, 69.0, 69.3, 69.8, 74.0, 78.3, 122.2, 127.4, 128.2, 128.6, 128.7, 135.7, 136.1, 145.3, 208.1; IR (ATR): υ = 3065, 3030, 2925, 2870, 1725, 1495, 1455 cm^{-1}; HRMS (ESI-TOF): calcd for *m/z* [M + Na]$^+$ $C_{46}H_{54}N_8NaO_8$: 869.3963; found: 869.3962; calcd for *m/z* [M + K]$^+$ $C_{46}H_{54}N_8KO_8$: 885.3702; found: 885.3697.

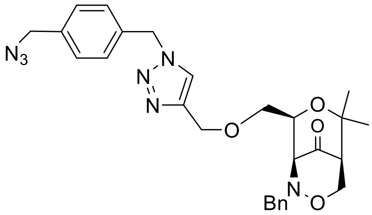

18

$[\alpha]_D^{22}$ = 66.0 (c = 0.15, MeOH); ^{1}H NMR ($CDCl_3$, 500 MHz): δ = 1.17, 1.38 (s, 3 H each), 2.30 (t, J = 2.0 Hz, 1 H), 3.18 (s, 1 H), 3.73–3.79, 3.85–3.92, 4.04–4.08 (3 m, 1 H, 2 H, 1 H), 4.12 (d, J = 13.5 Hz, 1 H), 4.34 (s, 2 H), 4.45–4.53 (m, 2 H), 4.55, 4.63 (2 d, J = 12.0 Hz, 1 H each), 5.40–5.45 (m, 2 H), 7.14–7.19 (m, 10 H); ^{13}C NMR ($CDCl_3$, 125 MHz): δ = 23.8, 26.7, 53.7, 54.3, 58.0, 64.9, 69.2, 69.4, 74.2, 78.4, 122.2, 127.5, 128.4, 128.5, 128.81, 128.85, 134.6, 136.1, 136.2, 145.6, the C=O signal could not be detected; IR (ATR): υ = 3060, 3030, 2925, 2870, 2095, 1725, 1495, 1455 cm^{-1}; HRMS (ESI-TOF): calcd for *m/z* [M + Na]$^+$ $C_{27}H_{31}N_7NaO_4$: 540.2335; found: 540.2331; calcd for *m/z* [M + K]$^+$ $C_{27}H_{31}N_7KO_4$: 556.2075; found: 556.2072.

1,3-Bis(1,2,3-triazole) 20: Following the **RP**, a mixture of 1,3-bis(bromomethyl)benzene (**8**) (26 mg, 0.099 mmol), compound **19** [3] (80 mg, 0.24 mmol), sodium azide (16 mg, 0.25 mmol), $CuSO_4 \cdot 5H_2O$ (5 mg, 0.02 mmol), sodium ascorbate (8 mg, 0.04 mmol), TBTA (11 mg, 0.022 mmol), L-proline (5 mg, 0.043 mmol), and Na_2CO_3 (4 mg, 0.038 mmol) in CH_3CN/H_2O (4:1, 0.5 mL) was stirred at 40 °C for 14 h. Standard work-up and purification by column chromatography (aluminum oxide, CH_2Cl_2/MeOH, 25:1) provided **20** (42 mg, 50%) as colorless liquid.

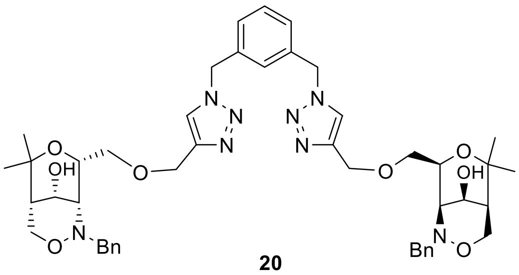

20

$[\alpha]_D^{22}$ = 24.5 (*c* = 1.5, MeOH); ^{1}H NMR ($CDCl_3$, 500 MHz): δ = 1.29, 1.52 (s, 6 H each), 1.59 (s_{br}, 2 H), 2.73 (s_{br}, 2 H), 3.67–3.73, 3.81–3.87, 3.98–4.07 (3 m, 2 H, 2 H, 4 H), 4.13 (d, *J* = 11.5 Hz, 2 H), 4.26 (d, *J* = 13.5 Hz, 2 H), 4.43 (t, *J* = 7.0 Hz, 2 H), 4.47, 4.61 (2 d, *J* = 12.0 Hz, 2 H each), 4.69 (t, *J* = 3.0 Hz, 2 H), 5.28, 5.34 (AB system, *J* = 15.5 Hz, 4 H), 5.66 (s_{br}, 1 H), 6.69 (s, 1 H), 7.05 (m_c, 2 H), 7.09–7.17, 7.20–7.28 (2 m, 4 H, 9 H), 7.34 (m_c, 1 H, Ar); ^{13}C NMR ($CDCl_3$, 125 MHz): δ = 26.5, 29.5, 42.3, 53.5, 57.3, 57.5, 64.3, 64.5, 66.6, 66.8, 70.8, 73.4, 122.5, 126.3, 127.0, 128.0, 128.2, 128.5, 129.7, 135.9, 138.0, 145.9; IR (ATR): υ = 3395, 2920, 2850, 1495, 1465, 1455 cm^{-1}; HRMS (ESI-TOF): calcd for *m/z* $[M + Na]^+$ $C_{46}H_{58}N_8NaO_8$: 873.4275; found: 873.4311; calcd for *m/z* $[M + K]^+$ $C_{46}H_{58}N_8KO_8$: 889.4041; found: 889.4054.

1,4-Bis(1,2,3-triazole) 21: Following the **RP**, a mixture of 1,4-bis(bromomethyl)benzene (**16**) (42 mg, 0.16 mmol), **19** (130 mg, 0.39 mmol), sodium azide (27 mg, 0.41 mmol), $CuSO_4 \cdot 5H_2O$ (8 mg, 0.032 mmol), sodium ascorbate (13 mg, 0.066 mg), TBTA (17 mg, 0.032 mmol), L-proline (7 mg, 0.061 mmol), and Na_2CO_3 (7 mg, 0.066 mg) in CH_3CN/H_2O (4:1, 0.5 mL) was stirred at 40 °C for 21 h. Standard work-up and purification column chromatography (aluminum oxide, CH_2Cl_2/MeOH, 25:1) afforded **21** (109 mg, 80%) as colorless solid (m.p. 108–110 °C).

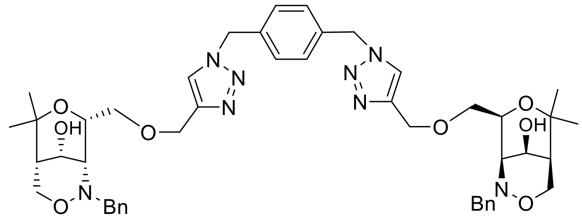

21

$[\alpha]_D^{22}$ = 74.5 (*c* = 1.0 MeOH); ^{1}H NMR ($CDCl_3$, 500 MHz): δ = 1.26, 1.46 (s, 6 H each), 1.53 (s, 2 H), 2.64 (s, 2 H), 3.55 (s, 2 H), 3.67–3.80, 3.92–4.02 (2 m, 4 H, 4 H), 4.08 (d, *J* = 11.5 Hz, 2 H), 4.23 (d, *J* = 13.5 Hz, 2 H), 4.37 (t, *J* = 6.0 Hz, 2 H), 4.51, 4.57 (2 d, *J* = 12.0 Hz, 2 H each), 4.62 (s, 2 H), 5.36 (s, 4 H), 7.10–7.16, 7.18–7.22, 7.25–7.29 (3 m, 6 H, 4 H, 2 H); ^{13}C NMR ($CDCl_3$, 125 MHz): δ = 26.5, 29.5, 42.5, 53.5, 57.2, 57.8, 64.2, 64.6, 66.2, 67.2, 71.4, 73.3, 122.6, 127.1, 128.2, 128.5, 128.7, 135.2, 137.8, 145.7; IR (ATR): υ = 3365, 3065, 3030, 2920, 2855, 1495, 1455 cm^{-1}; HRMS (ESI-TOF): calcd for *m/z* $[M + H]^+$ $C_{46}H_{59}N_8O_8$: 851.4416; found: 851.4475; calcd for *m/z* $[M + Na]^+$ $C_{46}H_{58}N_8NaO_8$: 873.4275; found: 873.4301.

(3*S*,4*S*,5*R*,6*S*)-5-Amino-3-(hydroxymethyl)-2,2-dimethyl-6-(propoxymethyl)tetrahydro-2*H*-pyran-4-ol (22): A stirred suspension of palladium on carbon (10%, 100 mg) in dry methanol (5 mL) was saturated with hydrogen for 30 min. Compound **19** (97 mg, 0.29 mmol) was added and the mixture was stirred under an atmosphere of hydrogen at room temperature for 17 h.

After filtration, the solution was concentrated under vacuum to give **22** (59 mg, 81%) as colorless liquid.

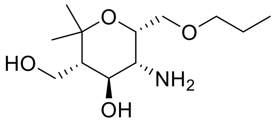

22

^{1}H NMR ($CDCl_3$, 500 MHz): δ = 0.88 (t, J = 7.0 Hz, 3 H), 1.11, 1.28 (2 s, 3 H each), 1.55 (m_c, 2 H), 1.75 (t, J = 12.0 Hz, 1 H), 2.98 (t, J = 5.0 Hz, 1 H), 3.35–3.41 (m, 2 H), 3.50–3.54 (m, 2 H), 3.56–3.60 (m, 1 H), 3.70–3.77 (m, 2 H), 4.02–4.06 (m, 1 H), 4.14 (s_{br}, 4 H); ^{13}C NMR ($CDCl_3$, 125 MHz): δ = 10.5, 22.7, 24.4, 26.5, 48.4, 56.9, 62.9, 68.2, 70.2, 73.4, 74.9, 75.0;.HRMS (ESI-TOF): calcd for m/z $[M + H]^+$ $C_{12}H_{26}NO_4$: 248.1862; found: 248.1858.

(1*R*,5*S*,8*S*,9*S*)-2-Benzyl-8-{[(1-benzyl-1*H*-1,2,3-triazol-4-yl)methoxy]methyl}-6,6-dimethyl-3,7-dioxa-2-azabicyclo[3.3.1]nonan-9-ol (23): To a solution of **7** (40 mg, 0.09 mmol) in 3 mL of ethanol was added $NaBH_4$ (7 mg, 0.18) at 0 °C. The mixture was stirred for 3 h at room temperature. After removal of ethanol, water was added to the residue and the mixture was extracted with dichloromethane (3 × 20 mL). The combined organic phases were dried (Na_2SO_4), filtered and concentrated. Purification by column chromatography (aluminum oxide, hexanes/ethyl acetate, 5:1) gave **23** (40 mg, 99%) as colorless liquid.

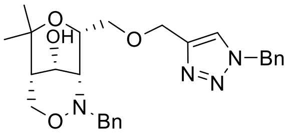

23

$[\alpha]_D^{22}$ = 49.0 (c = 1.5, MeOH); ^{1}H NMR ($CDCl_3$, 500 MHz): δ = 1.28, 1.48 (2 s, 3 H each), 1.57 (s, 1 H), 2.68 (s, 1 H), 3.14 (s, 1 H), 3.70–3.74, 3.78–3.82 (2 m, 1 H each), 4.00–4.07 (m, 2 H), 4.09–4.14 (m, 1 H), 4.25 (d, J = 13.5 Hz, 1 H), 4.40 (dt, J = 2.0, 6.0 Hz, 1 H), 4.52, 4.59 (AB system, J_{AB} = 12.0 Hz, 2 H), 4.65 (s, 1 H), 5.38–5.42 (m, 2 H), 7.12 (t, J = 7.5 Hz, 1 H), 7.17–7.36 (m, 10 H); ^{13}C NMR ($CDCl_3$, 125 MHz): δ = 26.5, 29.6, 42.5, 54.0, 57.3, 57.7, 64.5, 64.6, 66.4, 67.2, 71.3, 73.2, 122.4, 127.1, 128.0, 128.2, 128.5, 128.7, 129.0, 134.5, 137.8, 145.6; IR (ATR): υ = 3365, 3065, 3030, 2920, 2870, 1495, 1455 cm^{-1}; HRMS (ESI-TOF): calcd for m/z $[M + H]^+$ $C_{26}H_{33}N_4O_4$: 465.2502; found: 465.2468; calcd for m/z $[M + Na]^+$ $C_{26}H_{32}N_4NaO_4$: 487.2321; found: 487.2288; calcd for m/z $[M + K]^+$ $C_{26}H_{32}N_4KO_4$: 503.2061; found: 503.2028.

(3*S*,4*S*,5*R*,6*S*)-5-Amino-6-{[(1-benzyl-1*H*-1,2,3-triazol-4-yl)methoxy]methyl}-3-(hydroxymethyl)-2,2-dimethyltetrahydro-2*H*-pyran-4-ol (24): A stirred suspension of

palladium on carbon (10%, 50 mg) in methanol (4 mL) was saturated with hydrogen for 30 min. Compound **23** (12 mg, 0.026 mmol) was added and the mixture was stirred under an atmosphere of hydrogen at room temperature for 21 h. After filtration, the solution was concentrated under vacuum to give **24** (9 mg, 92%, estimated purity ca. 80%) as colorless liquid.

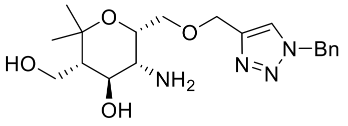

24

$[\alpha]_D^{22}$ = 10.2 (*c* = 0.35 MeOH); ^{1}H NMR (CD_3OD, 500 MHz): δ = 1.27, 1.48 (2 s, 3 H each, 1.60 (m_c, 1 H), 2.65 (m_c, 1 H), 3.67 (dd, *J* = 6.1, 9.3 Hz, 1 H), 3.75 (dd, *J* = 6.3, 9.3 Hz, 1 H), 4.05 (dd, *J* = 1.8, 12.4 Hz, 1 H), 4.08 (d, *J* = 13.4 Hz, 1 H), 4.24 (dd, *J* = 1.8, 12.4 Hz, 1 H), 4.28 (d, *J* = 13.4 Hz, 1 H), 4.35 (dt, *J* = 1.9, 6.2 Hz, 1 H), 4.51, 4.58 (AB system, J_{AB} = 12.3 Hz, 2 H), 4.64 (t, *J* = 3.5 Hz, 1 H, 4-H), 7.13–7.24, 7.29–7.38 (2 m, 2 H, 3 H), 7.79 (s, 2 H); ^{13}C NMR (CD_3OD, 100 MHz): δ = 26.2, 29.2, 43.2, 54.3, 53.2, 57.5, 58.3, 64.0, 64.5, 66.4, 68.1, 71.7, 74.3, 124.7, 127.6, 128.5, 128.6, 129.0, 129.3, 129.4, 136.1, 138.6, 145.9.

Reduction of 1,4-Bis(1,2,3-triazole) 21 to compound 25: Analogously to the reduction of compound **23**, palladium on carbon (10%, 21 mg) in dry methanol (1 mL) and compound **21** (22 mg, 0.026 mmol) gave after 5 d at room temperature, work-up and purification by column chromatography (aluminum oxide, CH_2Cl_2/MeOH, 5:1 to 1:3) provided **25** (7 mg, 40%, estimated purity ca. 80%) as colorless liquid.

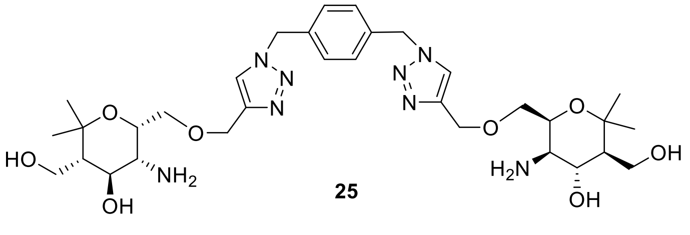

25

^{1}H NMR (CD_3OD, 500 MHz): δ = 1.21, 1.29 (2 s, 6 H each, 1.78–1.85 (m, 2 H), 2.67 (t, *J* = 5.5 Hz, 2 H), 3.49 (dd, *J* = 8.5, 11.0 Hz, 2 H), 3.64–3.72 (m, 4 H), 3.81 (dd, *J* = 5.0, 11.0 Hz), 4.00–4.07 (m, 4 H), 4.59, 4.65 (AB system, J_{AB} = 11.0 Hz, 4 H), 5.59 (s, 4 H), 7.34 (s, 4 H), 7.94 (s, 2 H); ^{13}C NMR (CD_3OD, 125 MHz): δ = 23.0, 26.5, 42.7, 49.4, 53.8, 62.5, 64.4, 66.5, 70.7, 71.3, 71.7, 75.8, 124.4, 128.7, 129.1, 129.5, 136.6, 146.1 (s, C-4´).

Samarium diiodide-promoted reduction of 1,4-bis(1,2,3-triazole) 24 to compound 26: 1,2-Diiodoethane (178 mg, 0.63 mmol) and samarium (103 mg, 0.69 mmol) were transferred into a dried flask under argon. THF (4 mL) was added under argon and the resulting solution was

stirred under argon [5]. After the solution turned blue, the mixture was stirred for further 2 h. To 2 mL of the solution was added **21** (40 mg, 0.047 mmol). The mixture stirred for 5 h at room temperature, then quenched with aqueous $NaHCO_3$ solution. After extraction of the mixture with dichloromethane, the organic phases were combined and dried (Na_2SO_4). After filtration and removal of solvent, the residue was purified by column chromatography (aluminum oxide, CH_2Cl_2/MeOH, 10:1) to give **26** (20 mg, 50%, estimated purity ca. 90%) as colorless liquid.

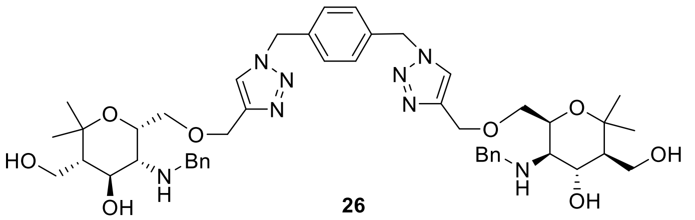

26

^{1}H NMR (CD_3OD, 500 MHz): δ = 1.30, 1.40 (2 s, 6 H each, 1.68–1.70 (m, 2 H), 3.02 (s, 2 H), 3.62–3.66 (m, 4 H), 3.70–3.75, 3.76–3.82 (2 m, 2 H each), 3.93 (dd, *J* = 4.0, 11.0 Hz, 2 H), 4.03, 4.15 (2 d, *J* = 13.0 Hz, 2 H each), 4.13–4.19 (m, 4 H), 4.23 (m_c, 2 H), 4.59, 4.60 (AB system, *J* = 12.1 Hz), 5.57 (s, 4 H), 7.22–7.27, 7.28–7.35 (2 m, 5 H, 9 H), 7.88 (s, 2 H); ^{13}C NMR (CD_3OD, 125 MHz): δ = 25.9, 26.8, 50.2, 53.2, 58.7, 61.8, 64.2, 65.3, 66.0, 69.9, 71.4, 75.0, 123.9, 128.3, 128.6, 128.7, 129.0, 135.4, 135.8, 144.2; HRMS (ESI-TOF): calcd for *m/z* $[M + H]^+$ $C_{46}H_{63}N_8O_8$: 855.4769; found: 855.4778; calcd. for *m/z* $[M + Na]^+$ $C_{46}H_{62}N_8NaO_8$: 877.4588; found: 877.4603.

3. References

[1] Zhu, W.; Ma, D. *Chem. Commun.* **2004**, 888–889. doi: 10.1039/B400878B

[2] Hoffmann, R. W.; Kemper, B.; Metternich, R.; Lehmeier, T. *Liebigs Ann. Chem.* **1985**, 2246–2260. doi: 10.1002/jlac.198519851115

[3] Al-Harrasi, A.; Pfrengle, F.; Prisyazhnyuk, V.; Yekta, S.; Koóš, P.; Reissig, H.-U. *Chem.- Eur. J.* **2009**, *15*, 11632–11641. doi: 10.1002/chem.200900996

[4] Chan, T. R.; Hilgraf, R.; Sharpless, K. B.; Fokin, V. V. *Org. Lett.* **2004**, *6*, 2853–2855. doi: 10.1021/ol0493094

[5] Wefelscheid, U. K.; Berndt, M.; Reissig, H.-U. *Eur. J. Org. Chem.* **2008**, 3635–3646. doi: 10.1002/ejoc.200800293

4. Copies of NMR spectra

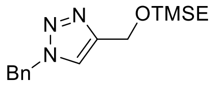

3

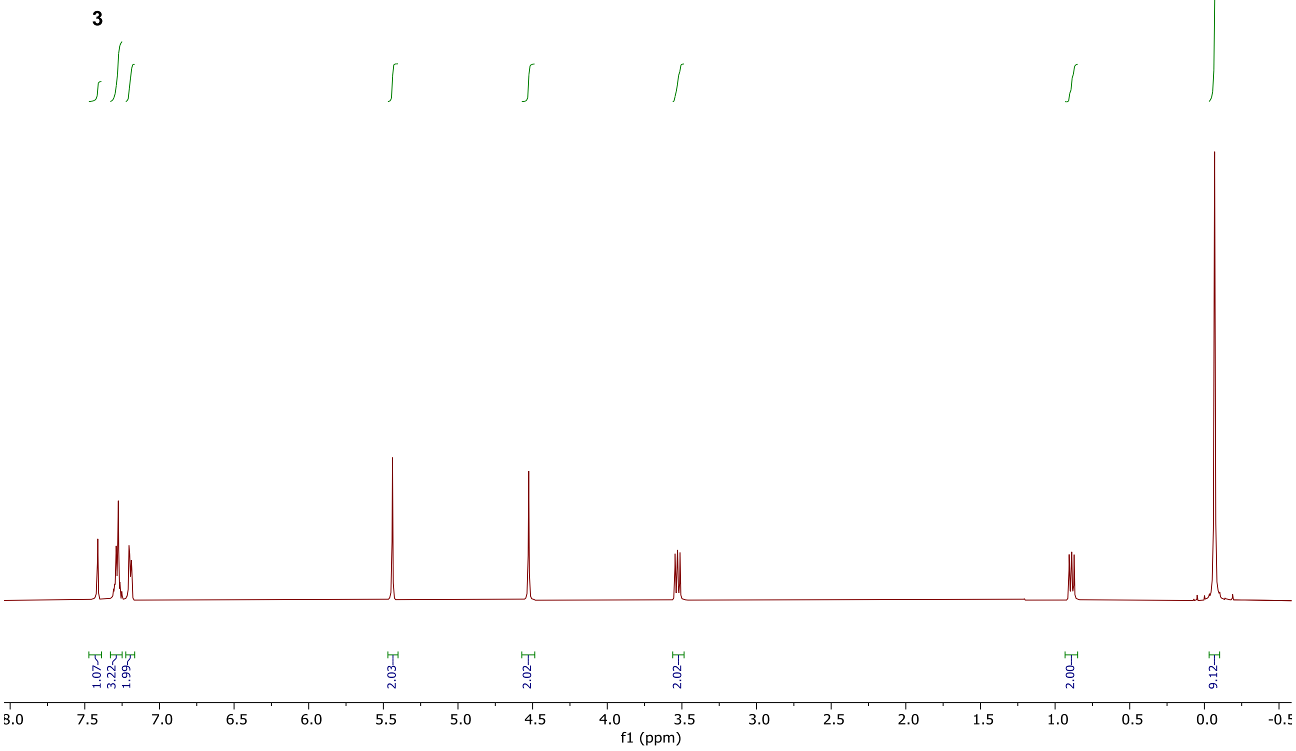

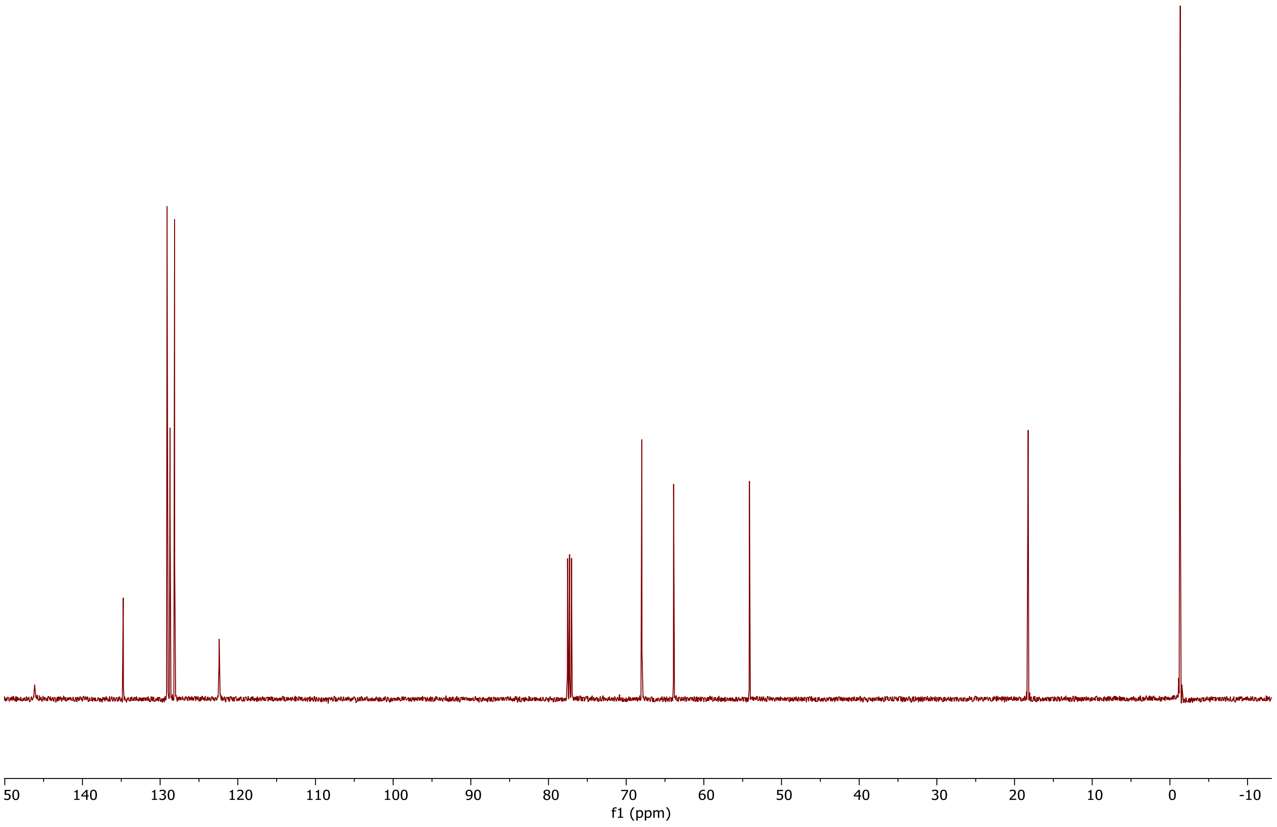

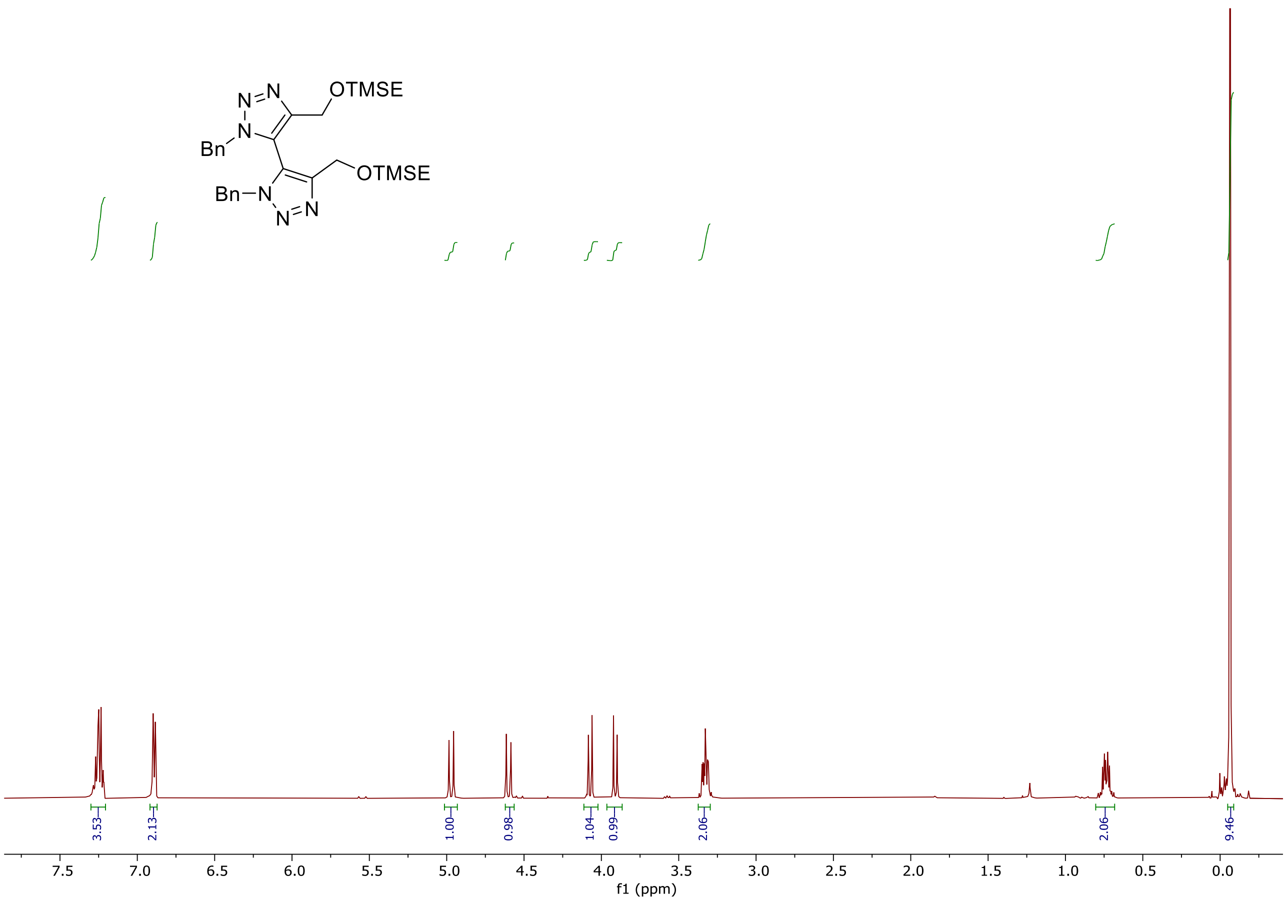
OTMSE
OTMSE
Bn
Bn
3.53
2.13
1.00
0.98
1.04
0.99
2.06
2.06
9.46
7.5 7.0 6.5 6.0 5.5 5.0 4.5 4.0 3.5 3.0 2.5 2.0 1.5 1.0 0.5 0.0
f1 (ppm)

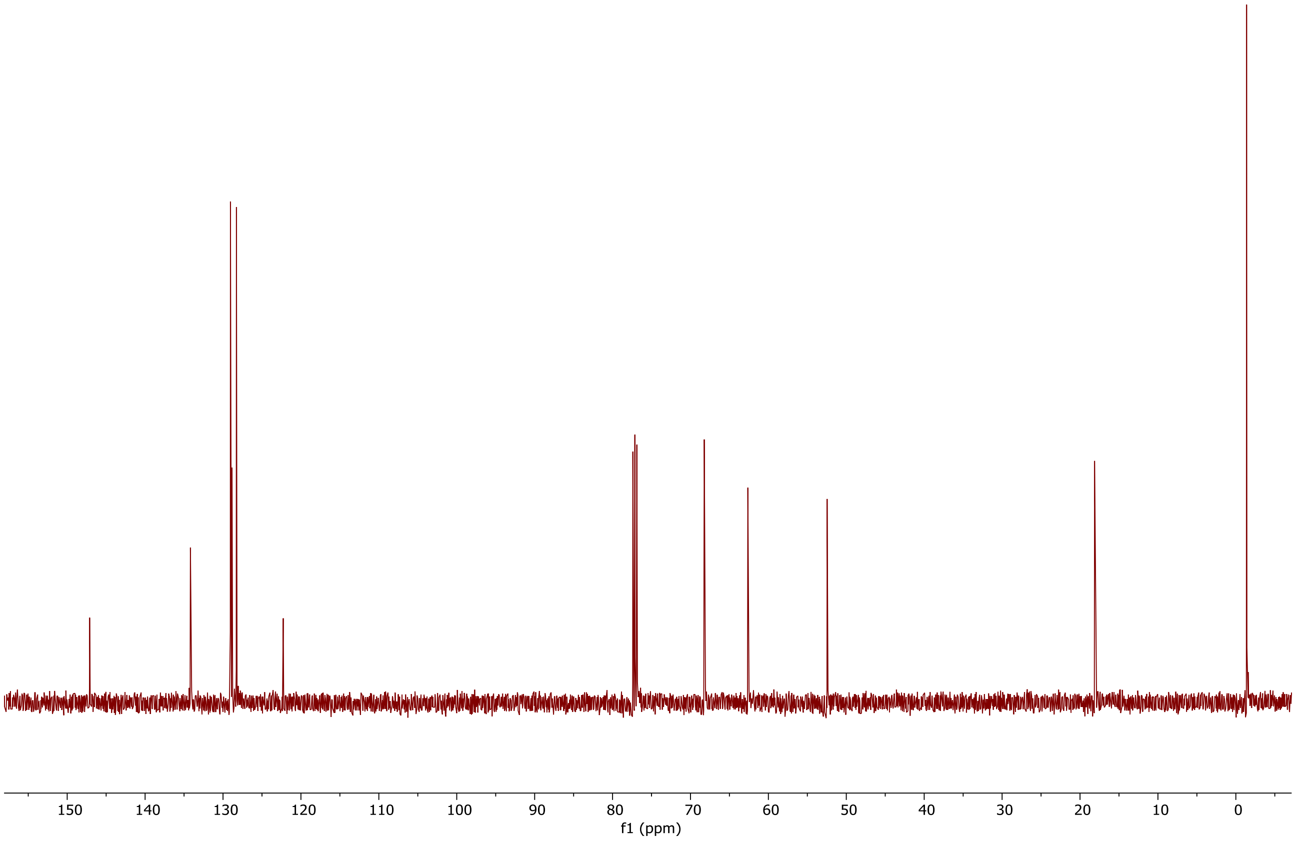
150 140 130 120 110 100 90 80 70 60 50 40 30 20 10 0
f1 (ppm)

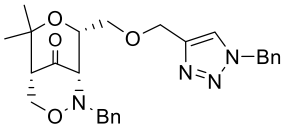

7

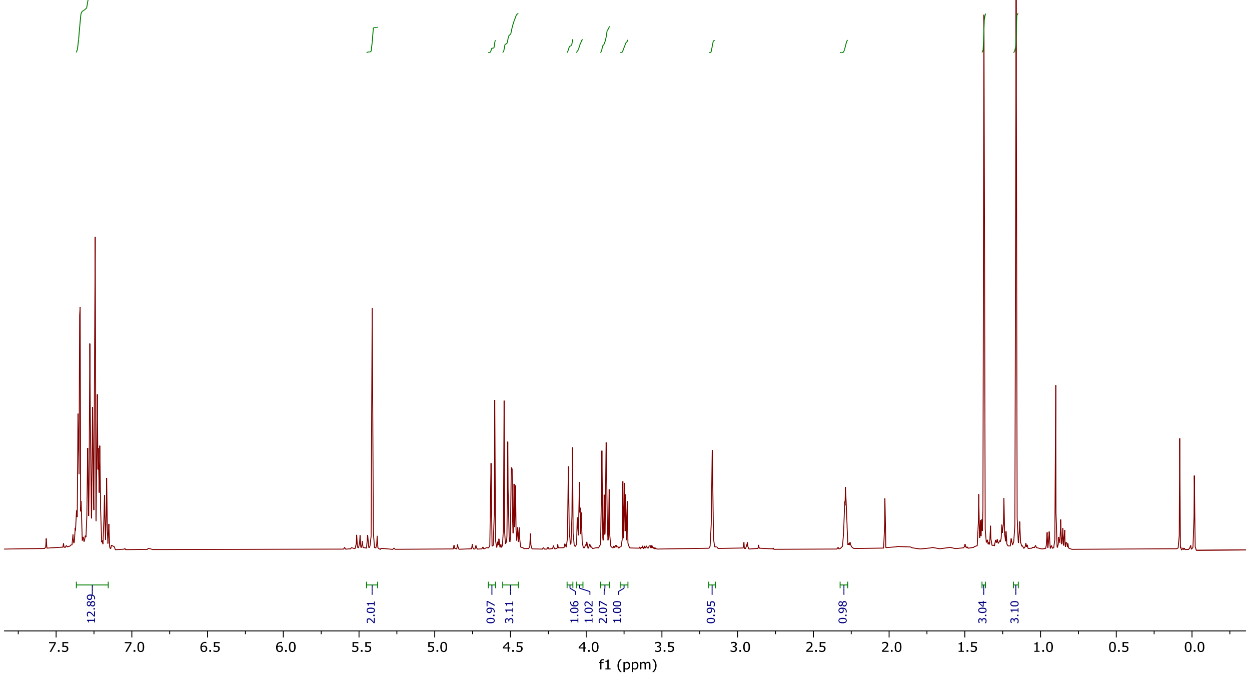
12.89
2.01
0.97
3.11
1.06
1.02
2.07
1.00
0.95
0.98
3.04
3.10
7.5 7.0 6.5 6.0 5.5 5.0 4.5 4.0 3.5 3.0 2.5 2.0 1.5 1.0 0.5 0.0
f1 (ppm)

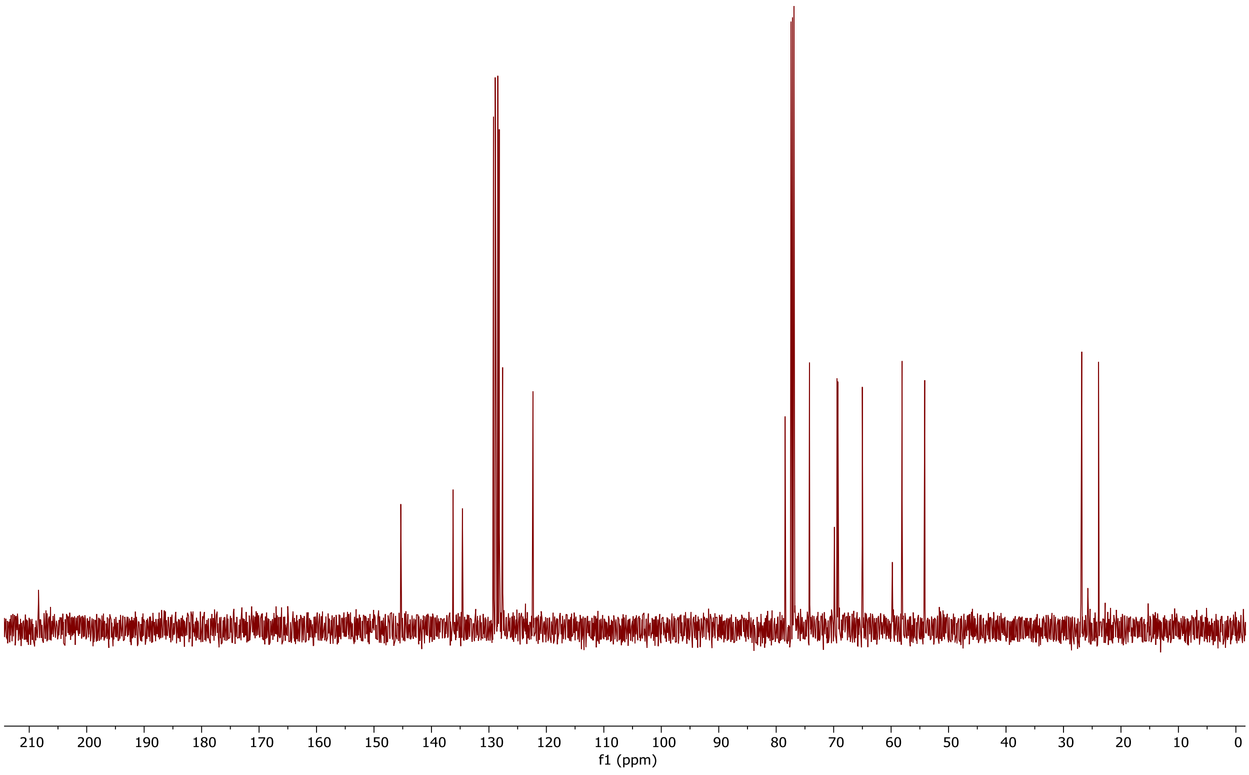
210 200 190 180 170 160 150 140 130 120 110 100 90 80 70 60 50 40 30 20 10 0
f1 (ppm)

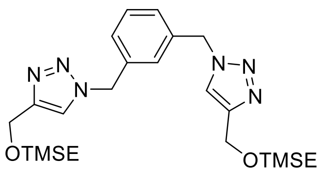
N=N
N
N–N
N
OTMSE
OTMSE

9

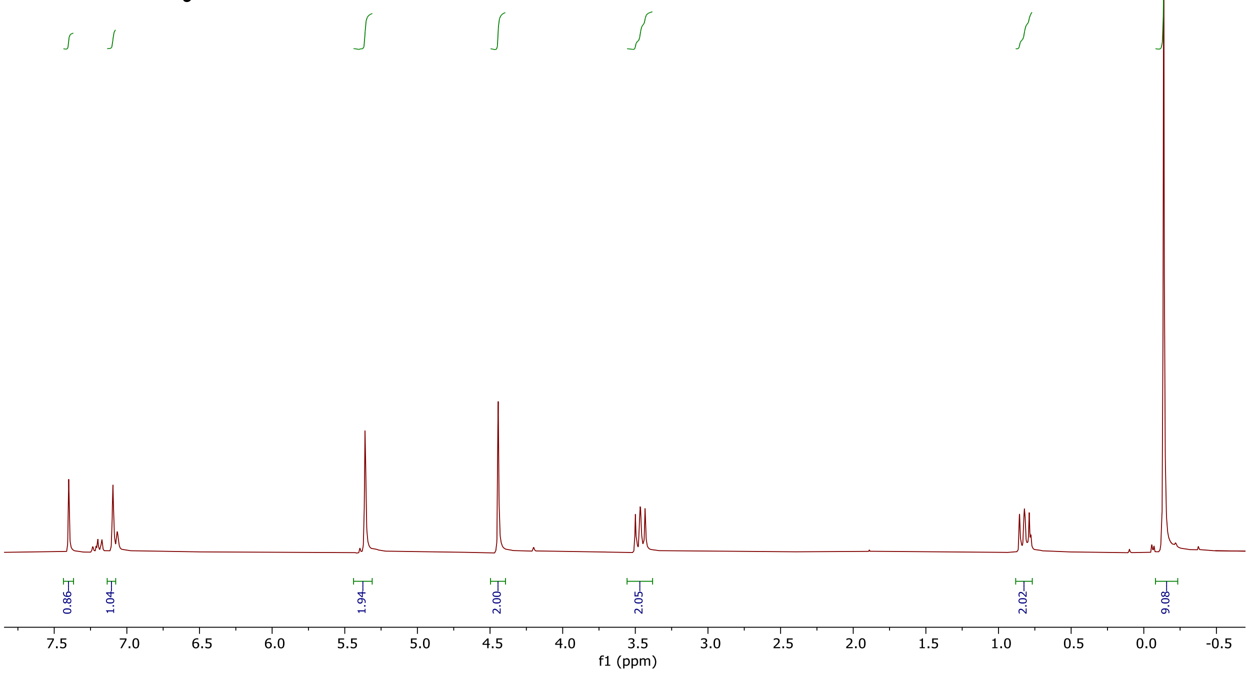
0.86
1.04
1.94
2.00
2.05
2.02
9.08
7.5 7.0 6.5 6.0 5.5 5.0 4.5 4.0 3.5 3.0 2.5 2.0 1.5 1.0 0.5 0.0 -0.5
f1 (ppm)

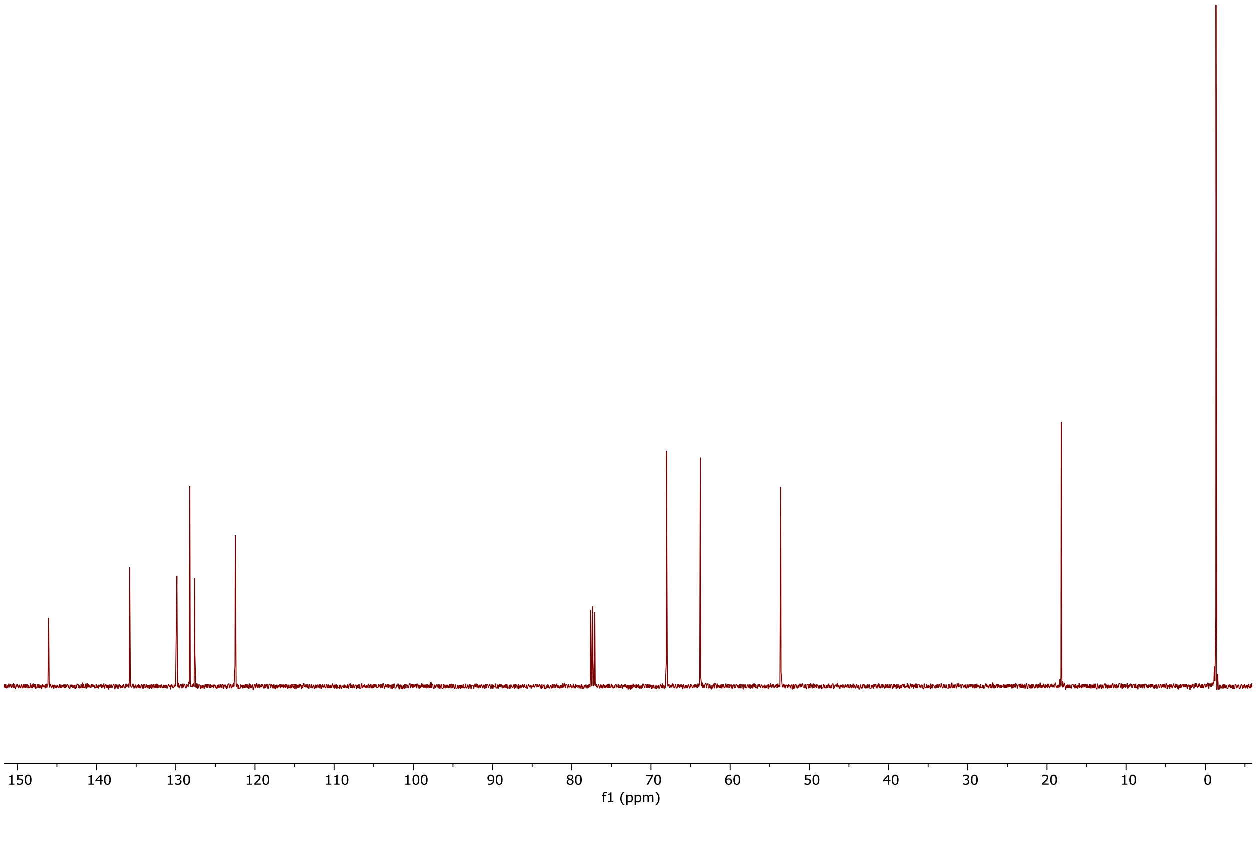
150 140 130 120 110 100 90 80 70 60 50 40 30 20 10 0
f1 (ppm)

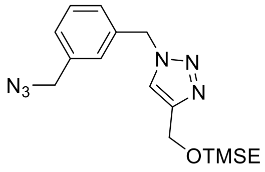
N3
N
N
N
OTMSE

10

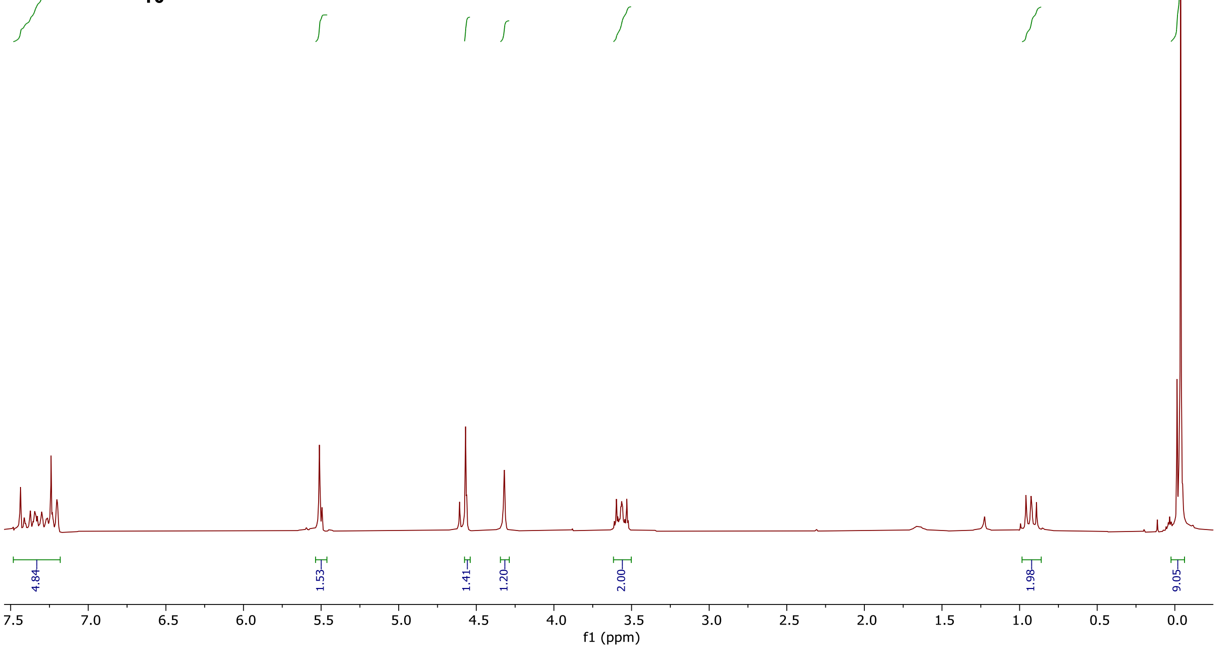
4.84
1.53
1.41
1.20
2.00
1.98
9.05
7.5 7.0 6.5 6.0 5.5 5.0 4.5 4.0 3.5 3.0 2.5 2.0 1.5 1.0 0.5 0.0
f1 (ppm)

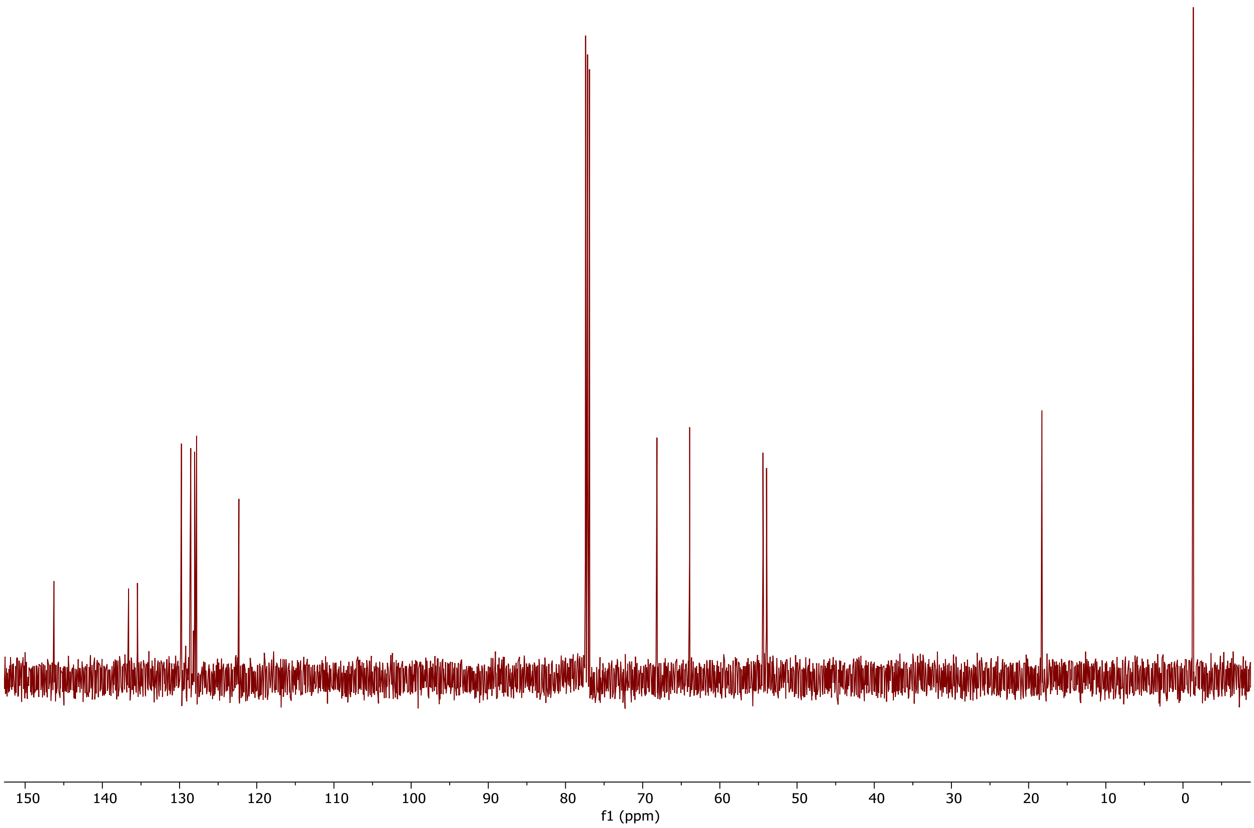
150 140 130 120 110 100 90 80 70 60 50 40 30 20 10 0
f1 (ppm)

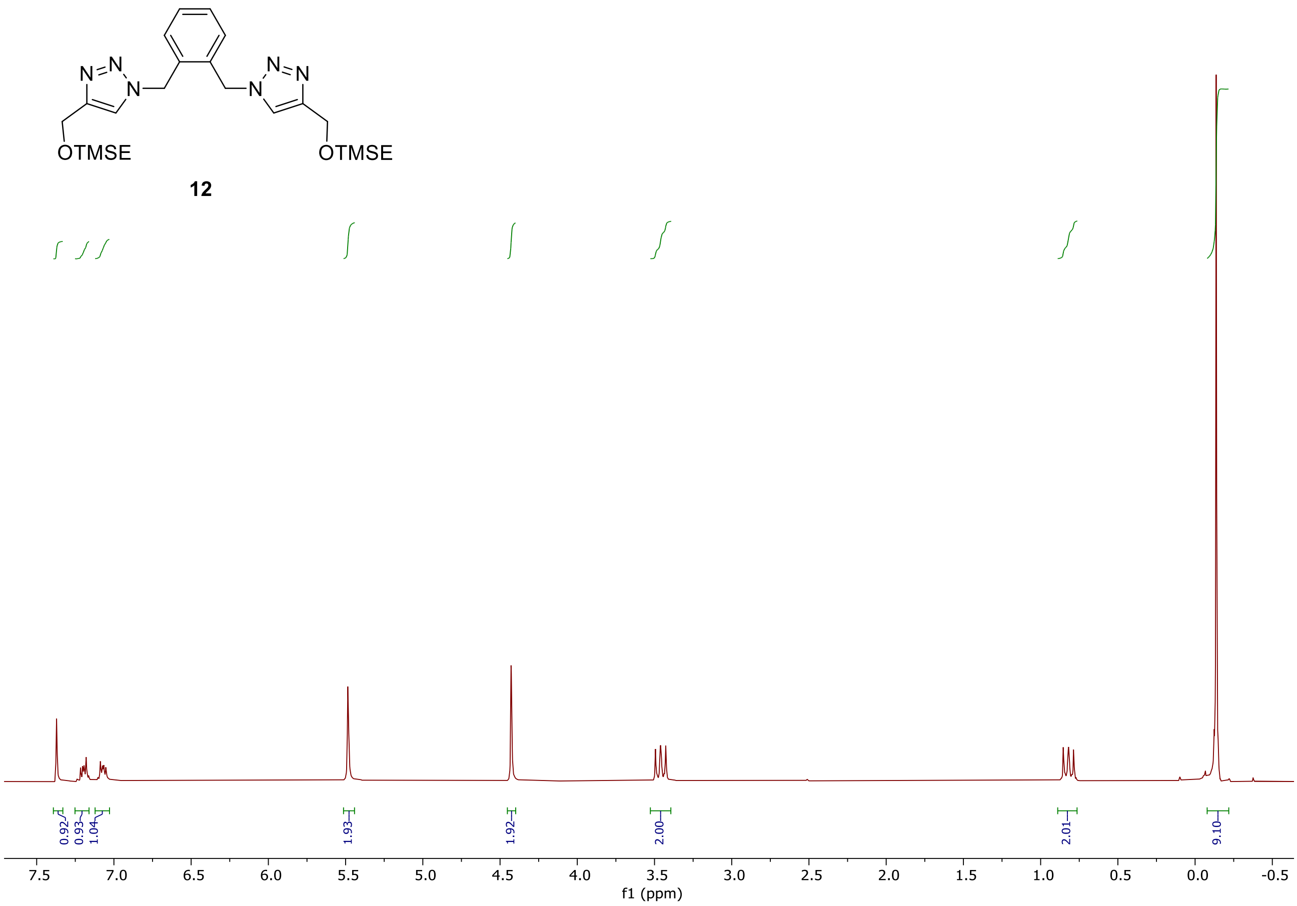
OTMSE
OTMSE
0.92
0.93
1.04
1.93
1.92
2.00
2.01
9.10
f1 (ppm)

12

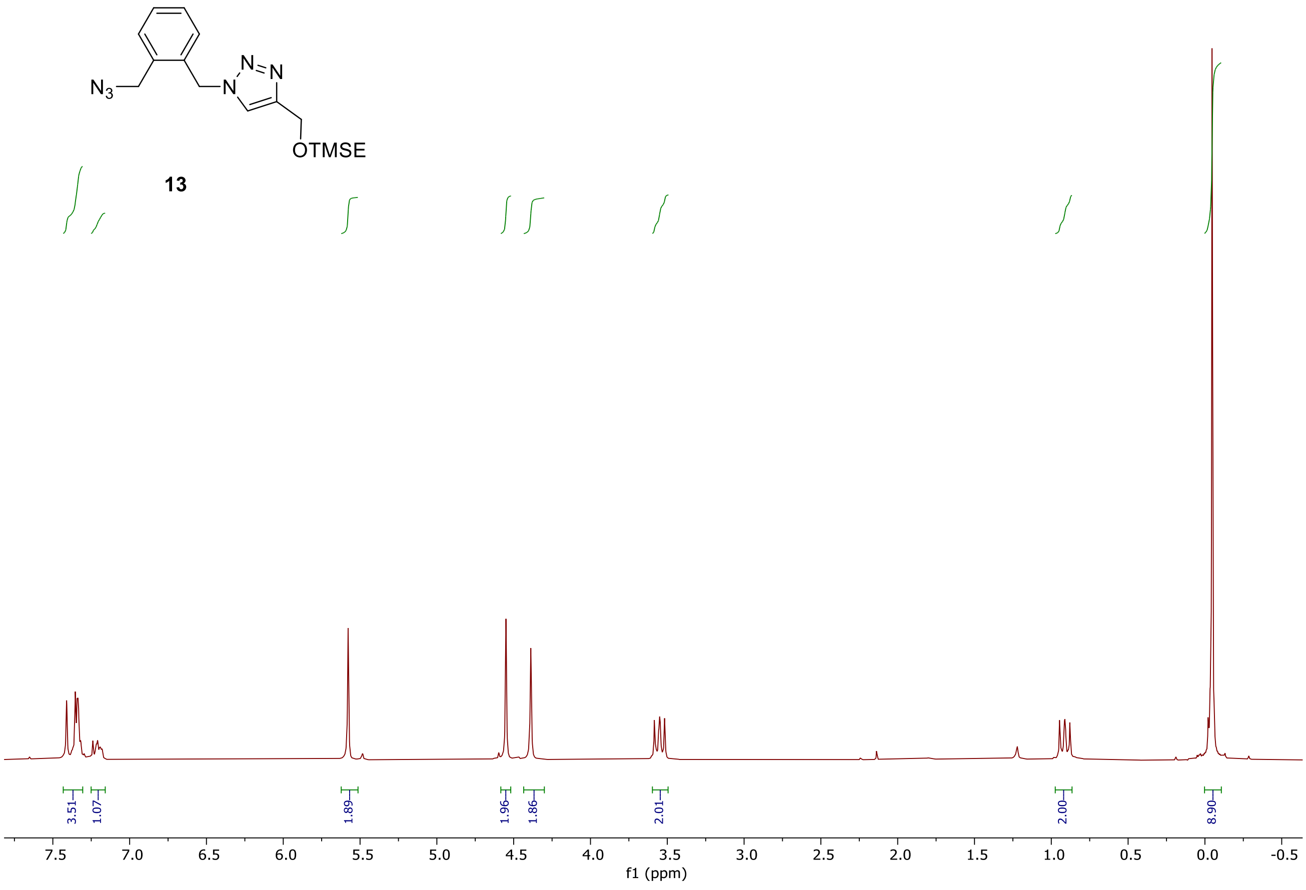
N3
OTMSE
3.51
1.07
1.89
1.96
1.86
2.01
2.00
8.90
f1 (ppm)

13

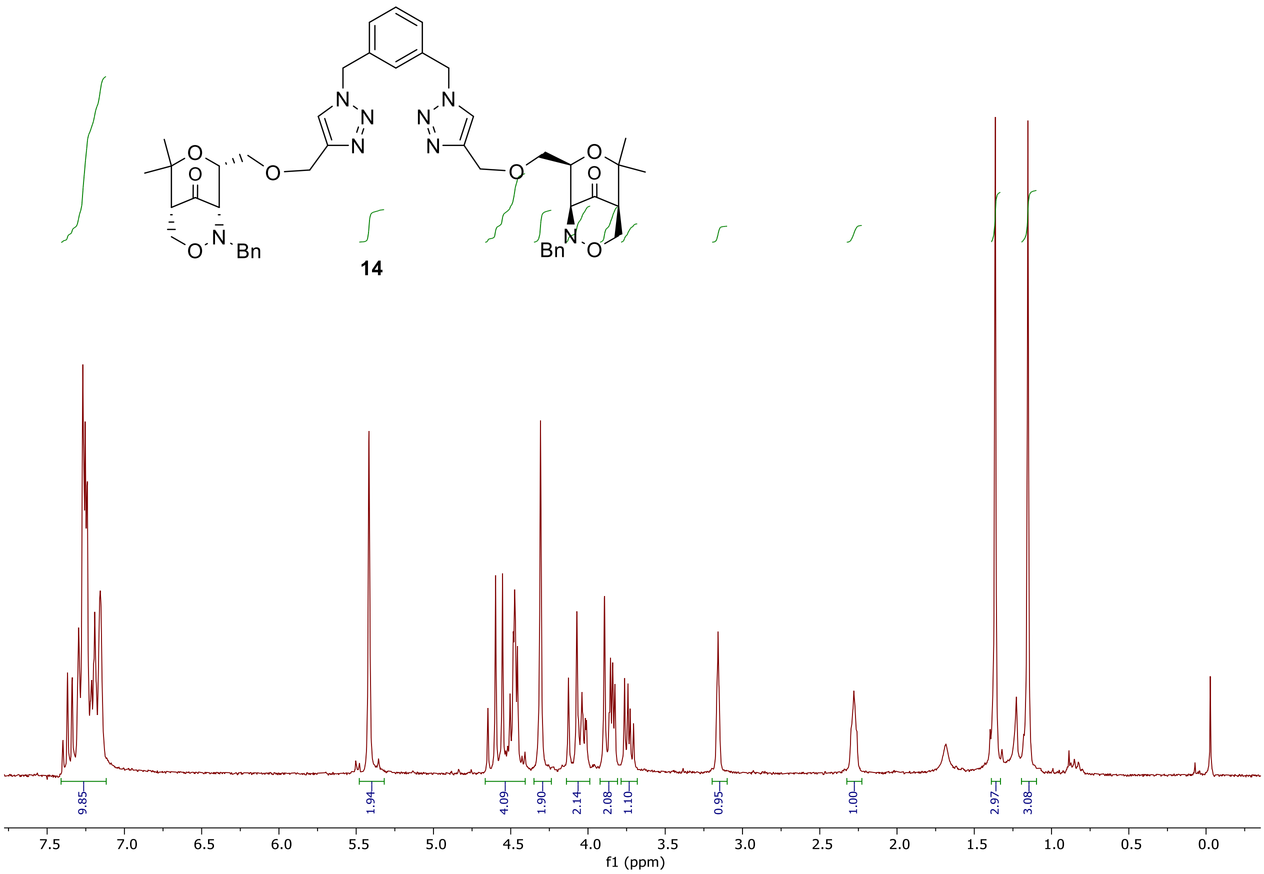
O
O
O
N
Bn
O
N
N
N
N
N
N
N
N
O
O
O
O
N
Bn
O
14
9.85
1.94
4.09
1.90
2.14
2.08
1.10
0.95
1.00
2.97
3.08
7.5 7.0 6.5 6.0 5.5 5.0 4.5 4.0 3.5 3.0 2.5 2.0 1.5 1.0 0.5 0.0
f1 (ppm)

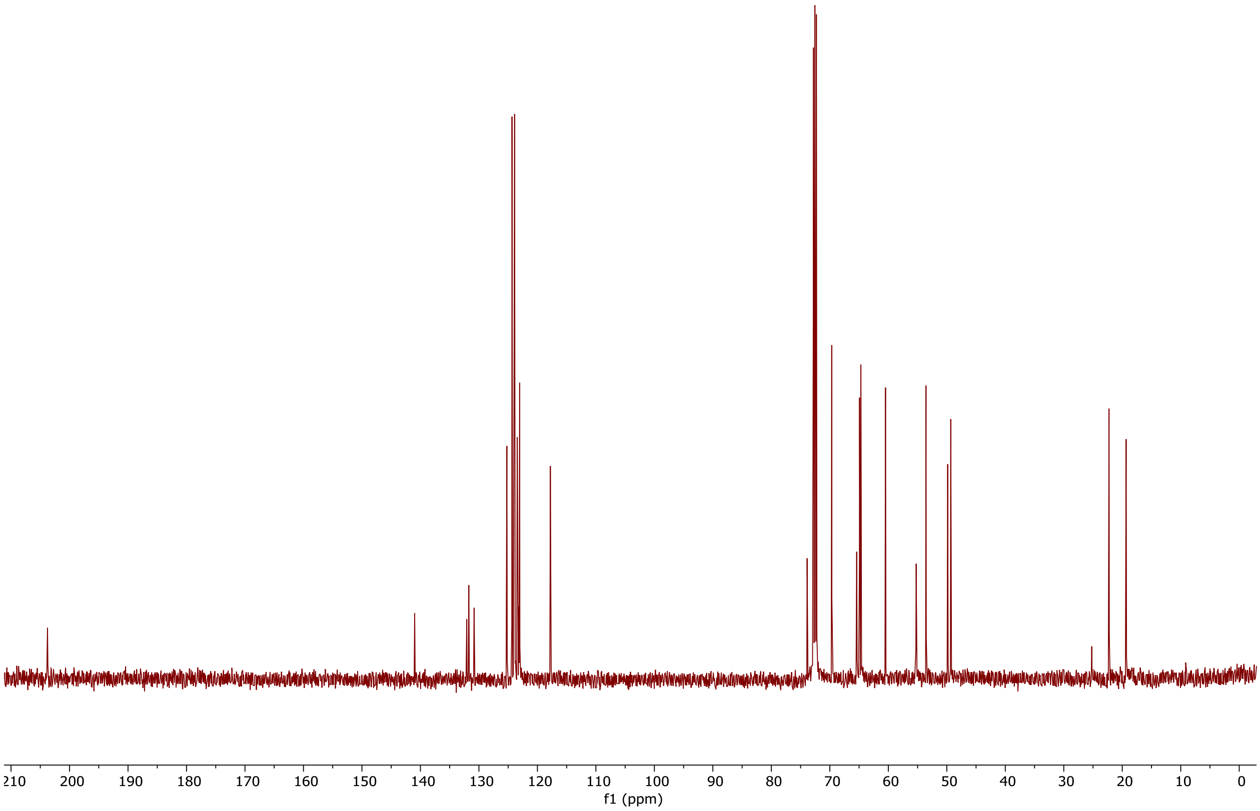
210 200 190 180 170 160 150 140 130 120 110 100 90 80 70 60 50 40 30 20 10 0
f1 (ppm)

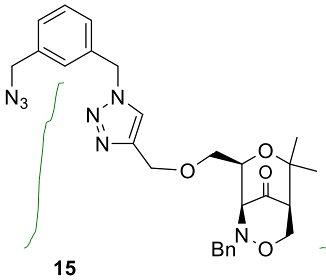
N3
N
N
N
O
O
O
N
Bn
O

15

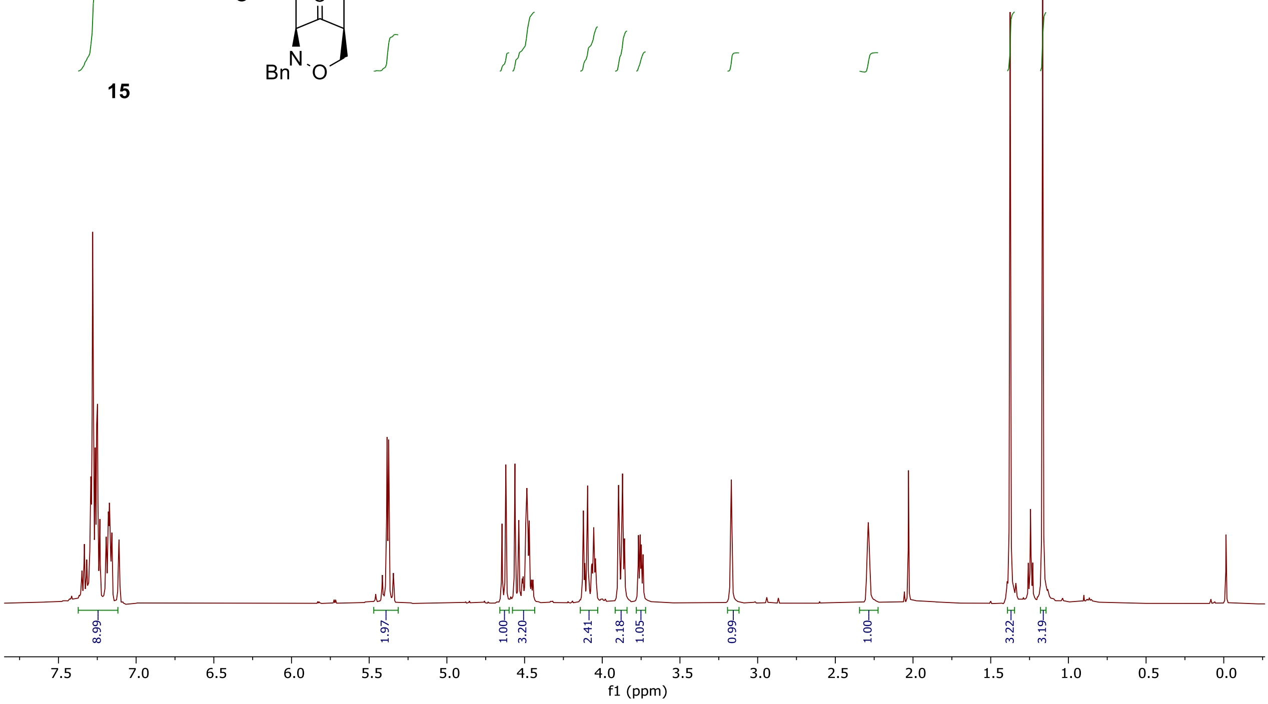
8.99
1.97
1.00
3.20
2.41
2.18
1.05
0.99
1.00
3.22
3.19
7.5
7.0
6.5
6.0
5.5
5.0
4.5
4.0
3.5
3.0
2.5
2.0
1.5
1.0
0.5
0.0
f1 (ppm)

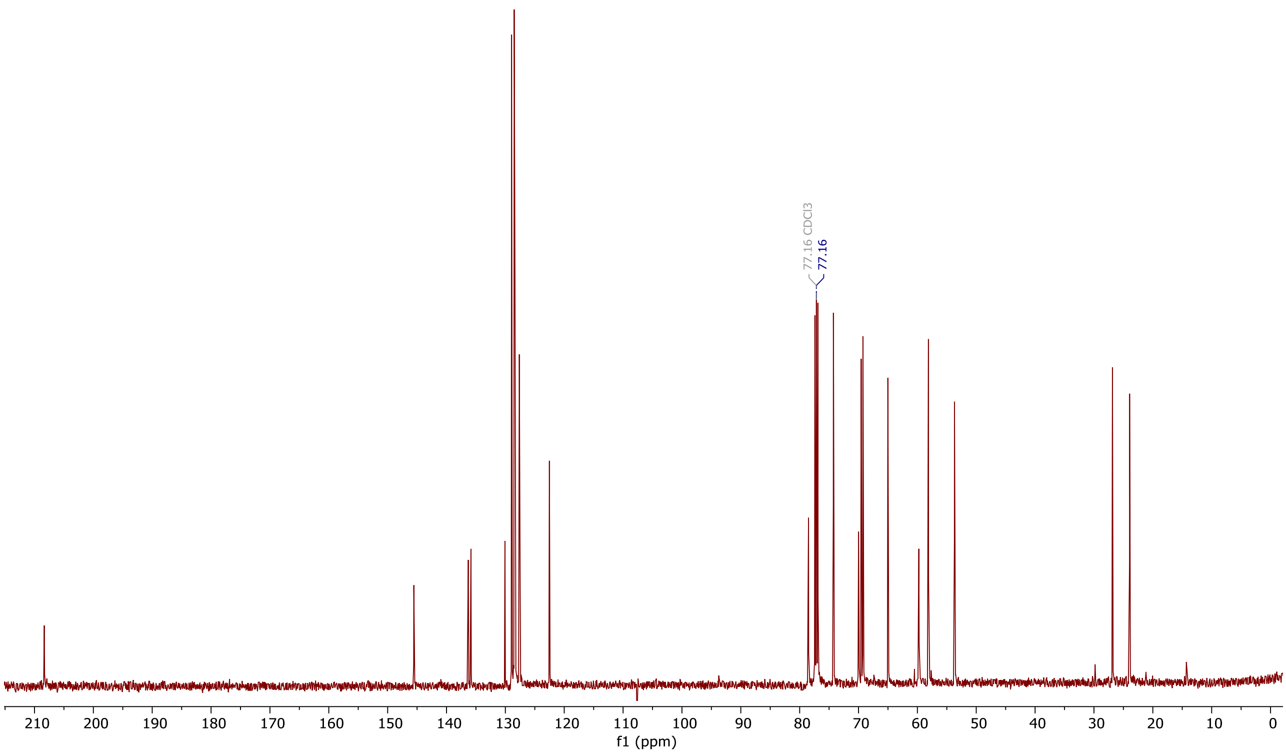
77.16 CDCl3
77.16
210
200
190
180
170
160
150
140
130
120
110
100
90
80
70
60
50
40
30
20
10
0
f1 (ppm)

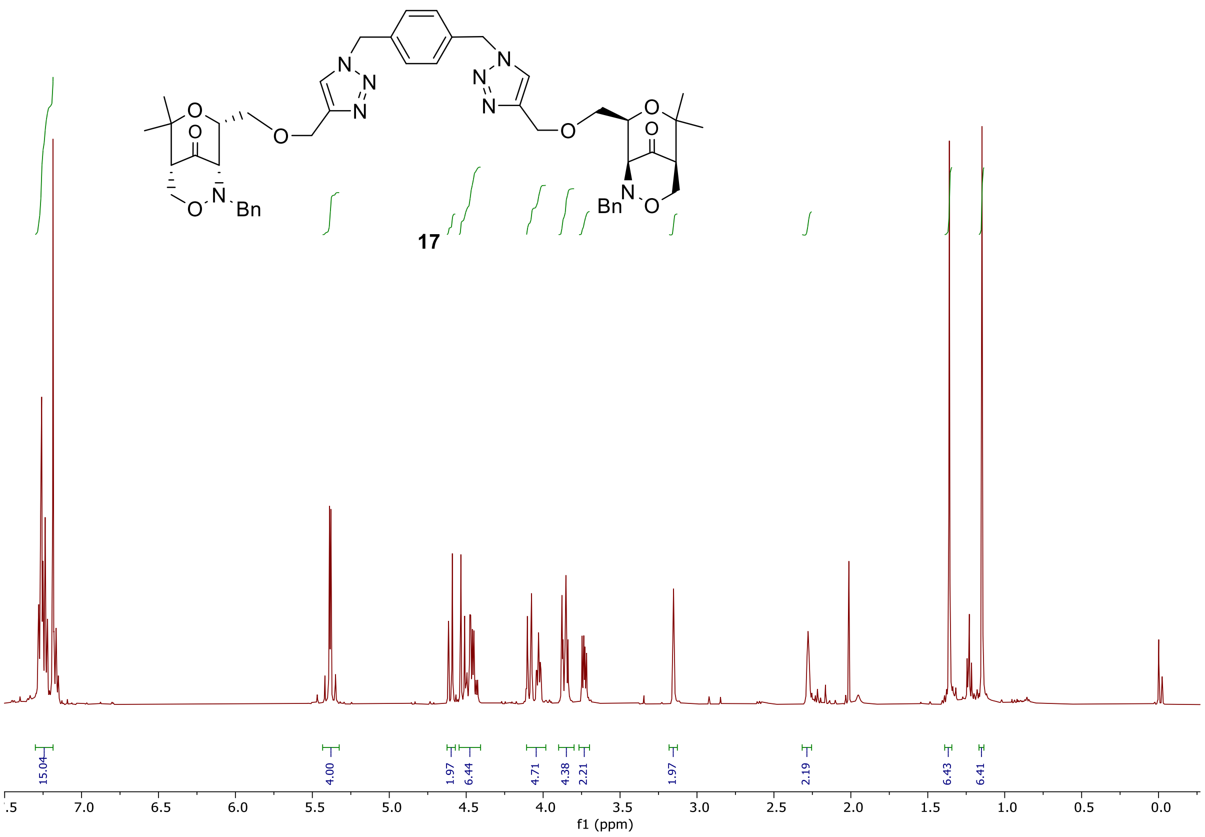
17
15.04
4.00
1.97
6.44
4.71
4.38
2.21
1.97
2.19
6.43
6.41
f1 (ppm)

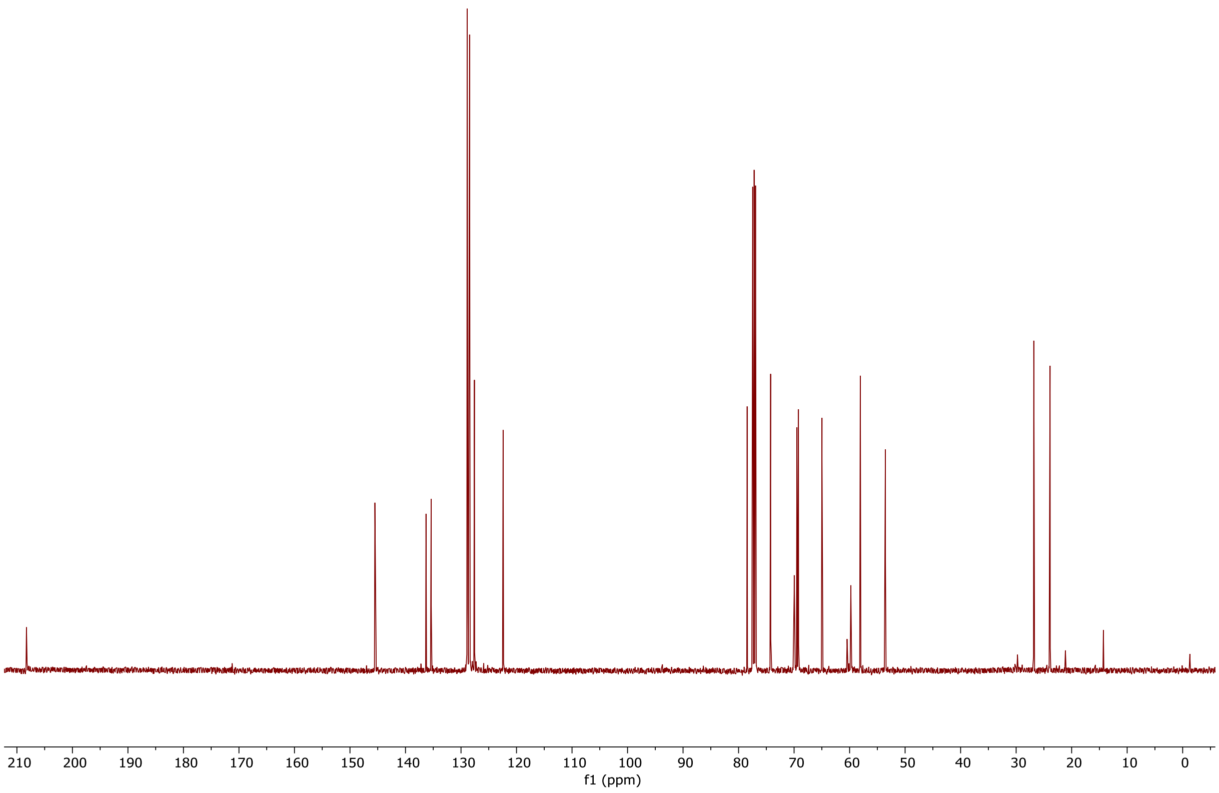
f1 (ppm)

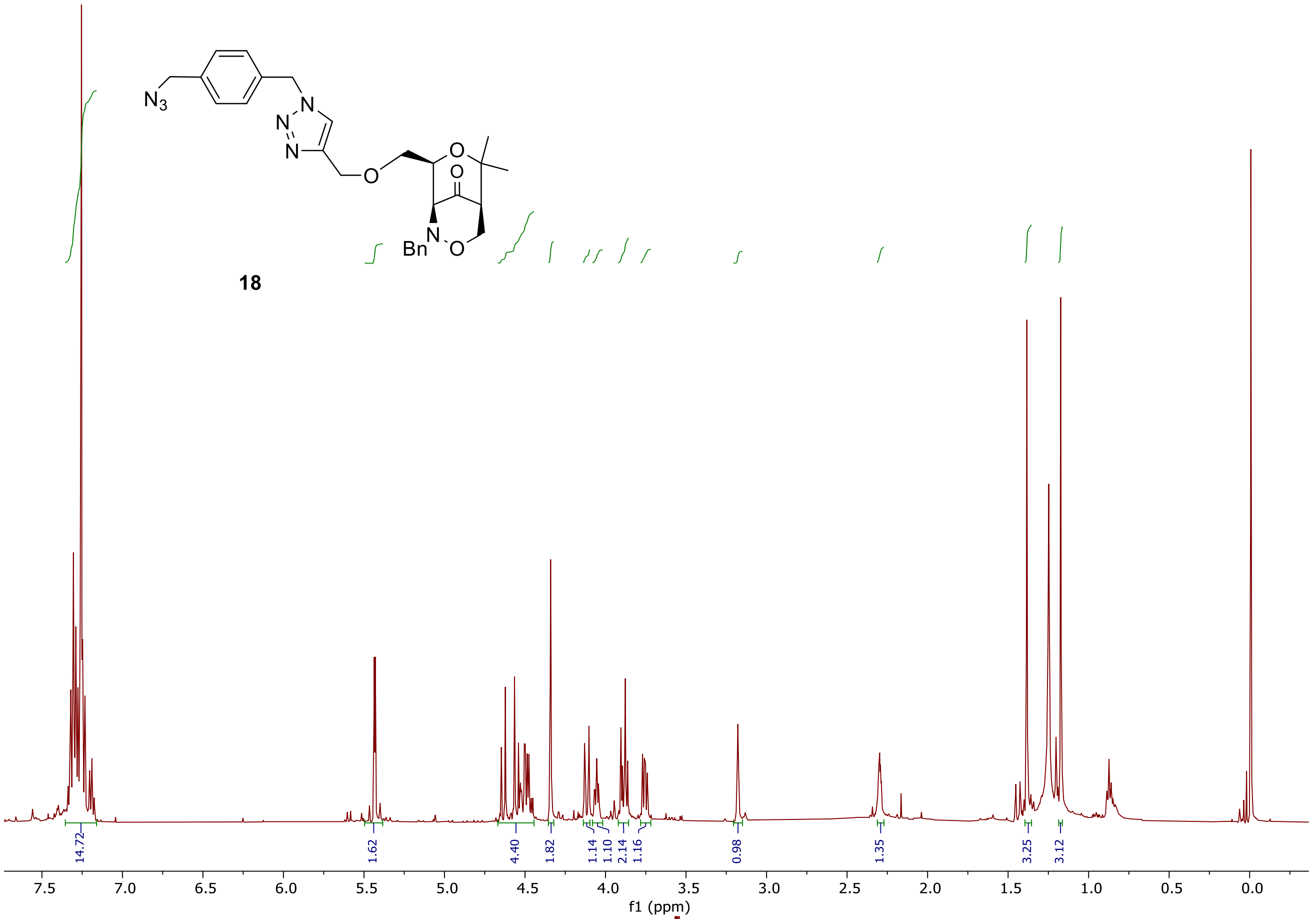

N_3
Bn
18
14.72
1.62
4.40
1.82
1.14
1.10
2.14
1.16
0.98
1.35
3.25
3.12
7.5 7.0 6.5 6.0 5.5 5.0 4.5 4.0 3.5 3.0 2.5 2.0 1.5 1.0 0.5 0.0
f1 (ppm)

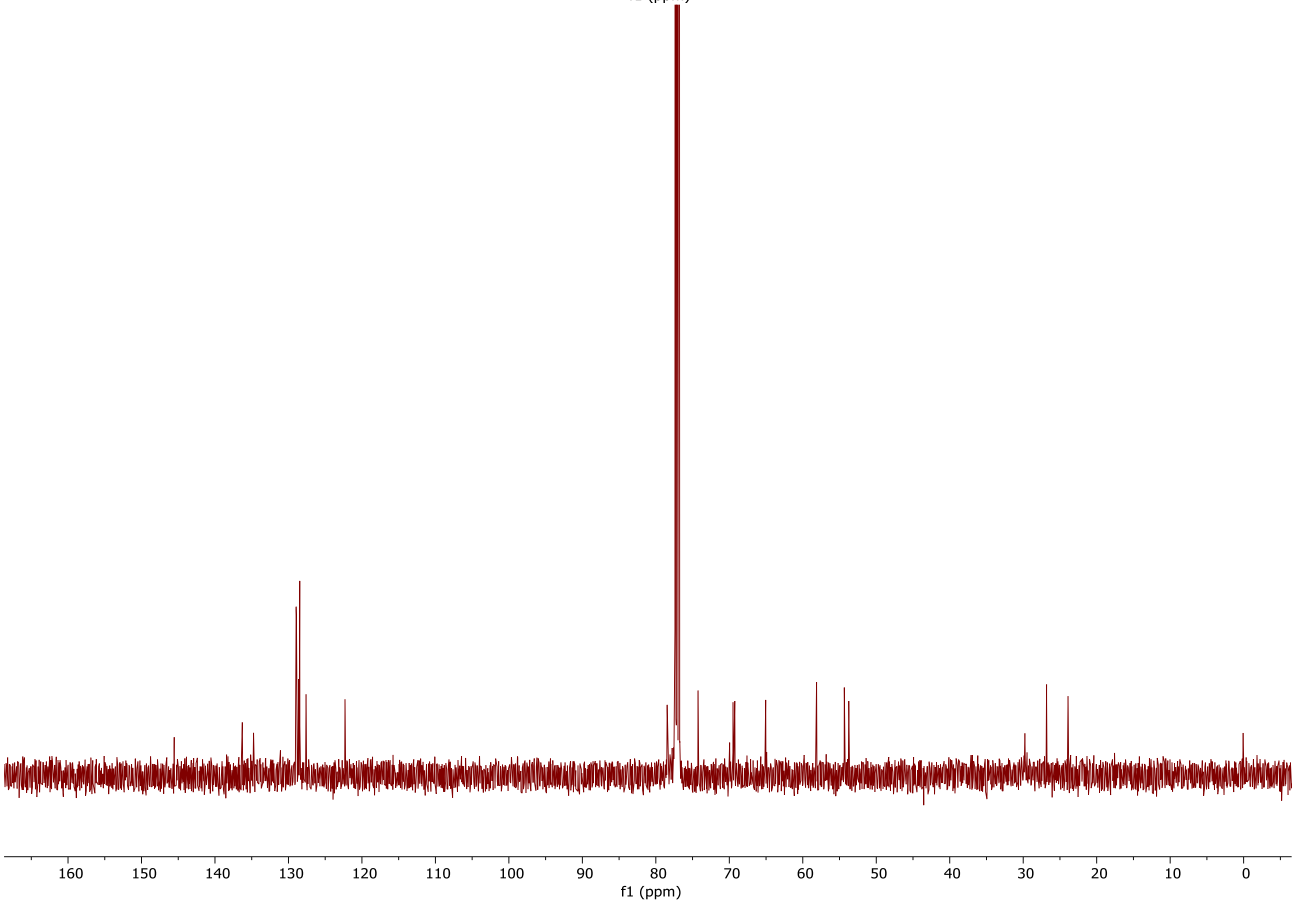

160 150 140 130 120 110 100 90 80 70 60 50 40 30 20 10 0
f1 (ppm)

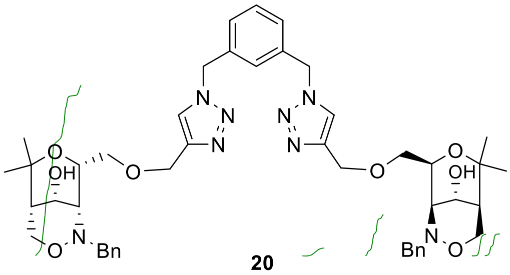
OH
O
N
Bn
OH
O
N
Bn

20

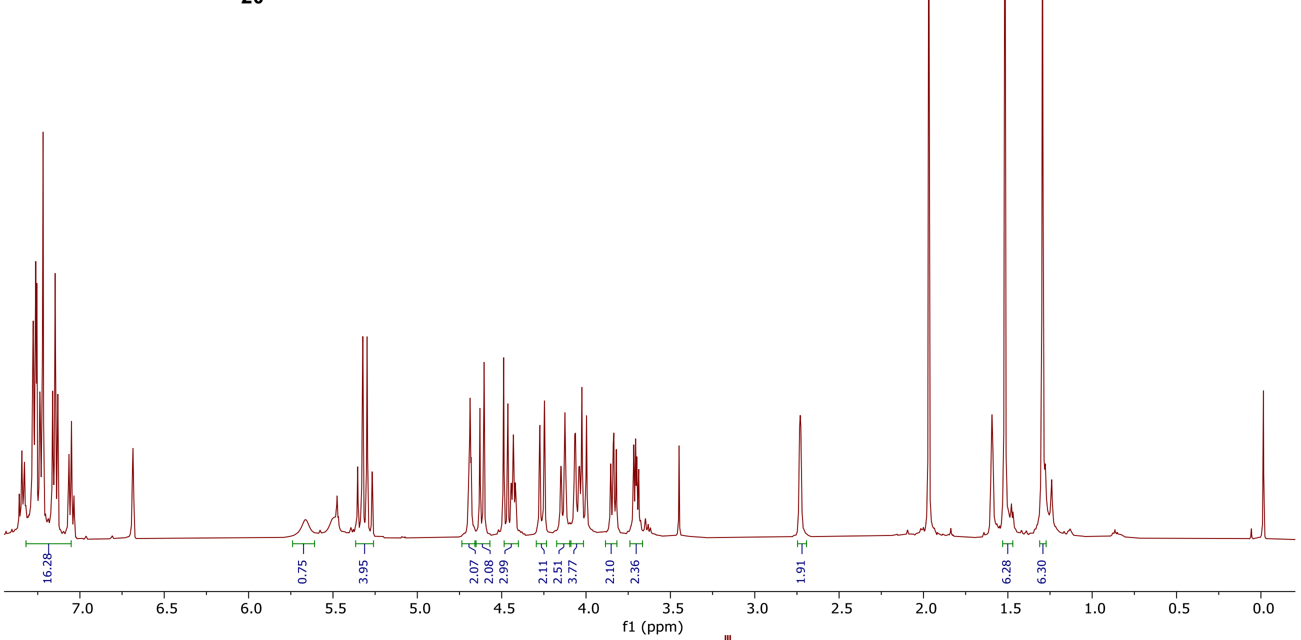
16.28
0.75
3.95
2.07
2.08
2.99
2.11
2.51
3.77
2.10
2.36
1.91
6.28
6.30
7.0
6.5
6.0
5.5
5.0
4.5
4.0
3.5
3.0
2.5
2.0
1.5
1.0
0.5
0.0
f1 (ppm)

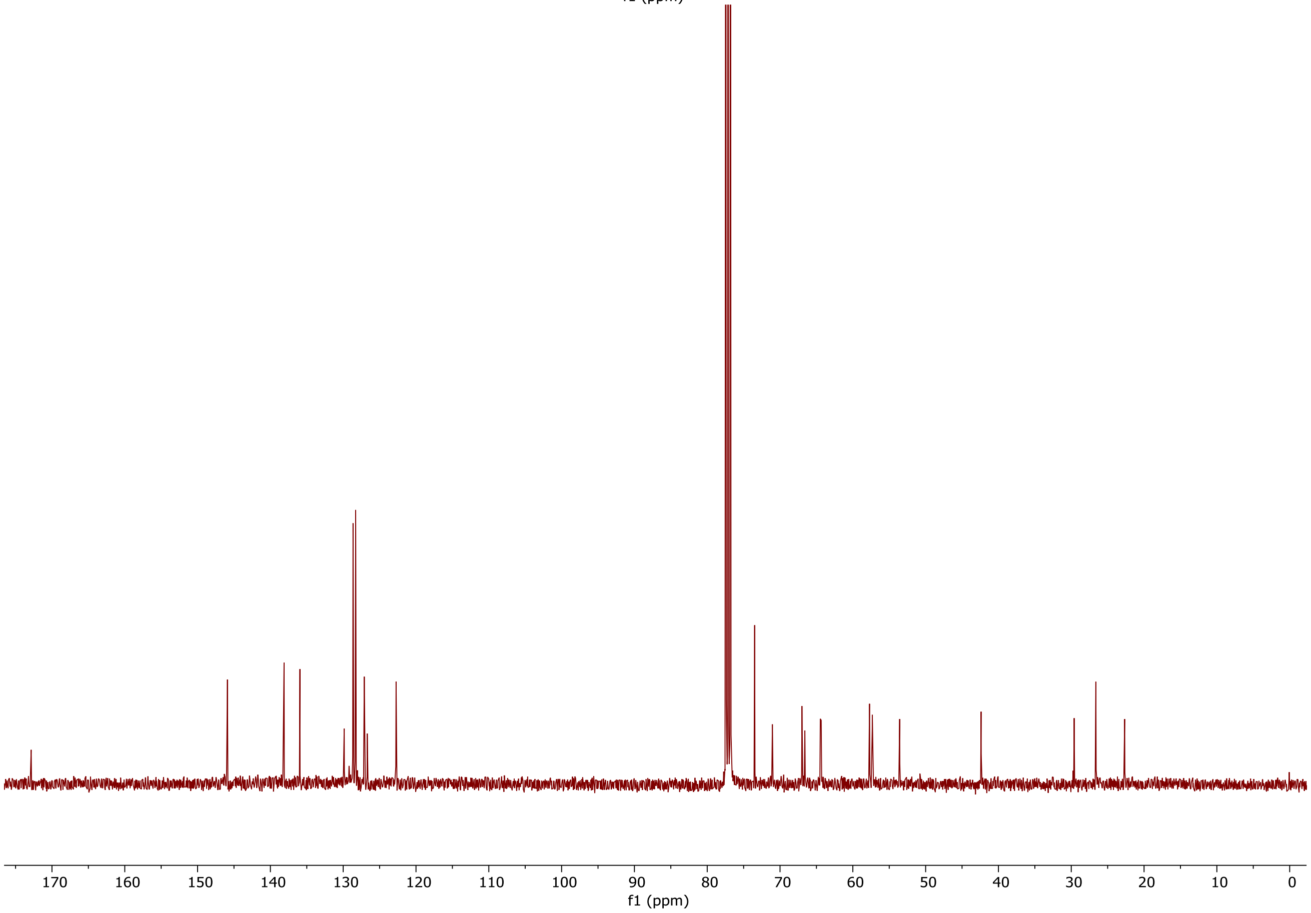
170
160
150
140
130
120
110
100
90
80
70
60
50
40
30
20
10
0
f1 (ppm)

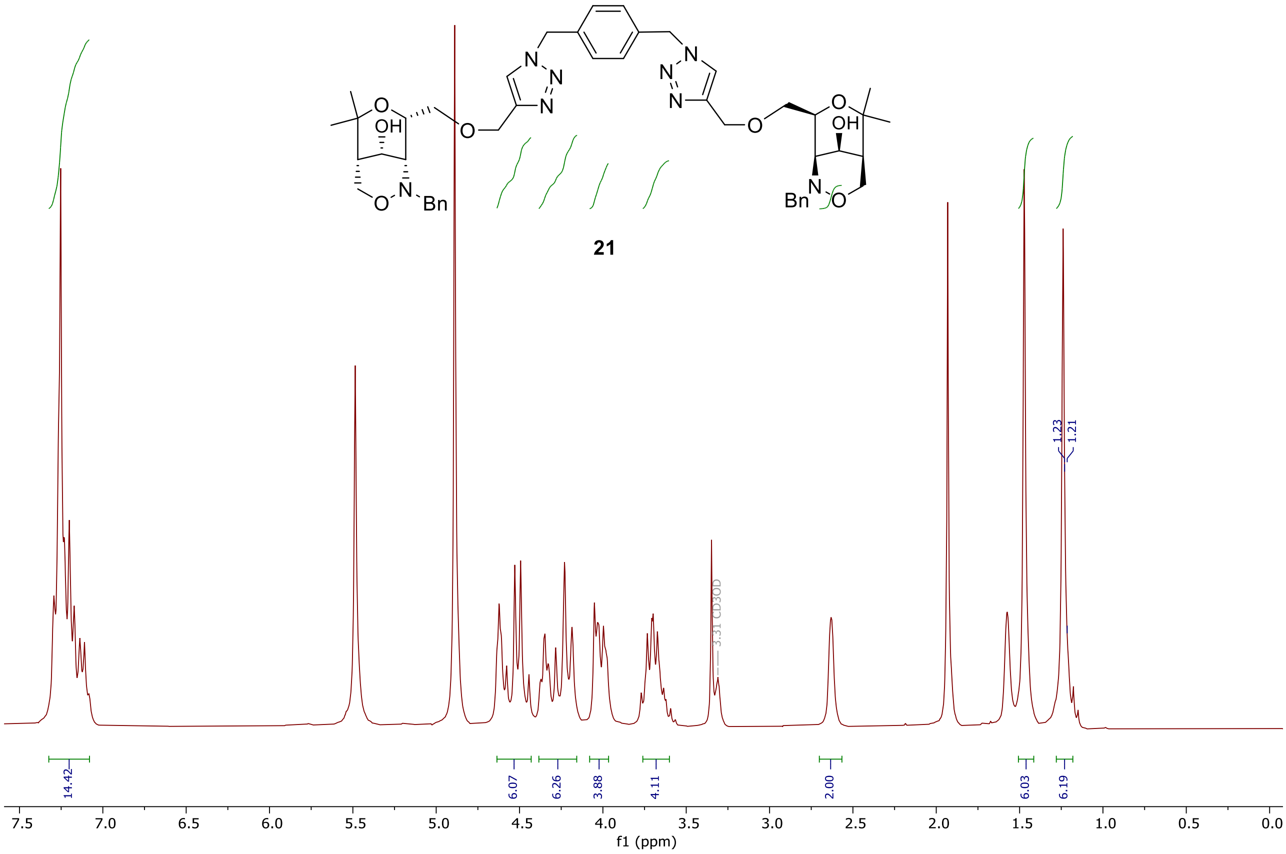

21
OH
N
Bn
O
3.31 CD3OD
1.23
1.21
14.42
6.07
6.26
3.88
4.11
2.00
6.03
6.19
f1 (ppm)

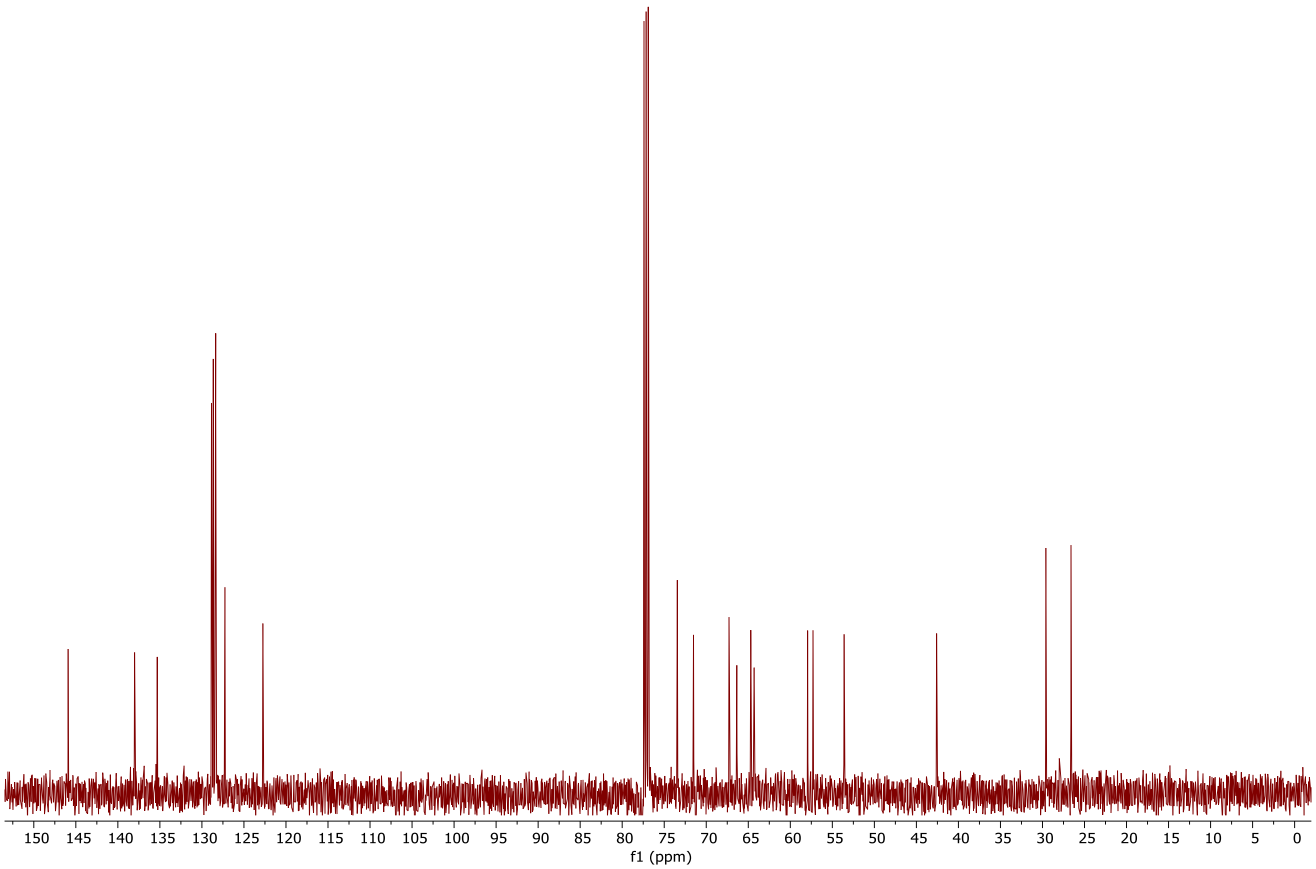

f1 (ppm)

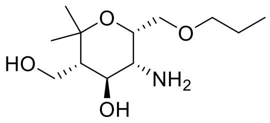
HO
O
O
NH2
OH

22

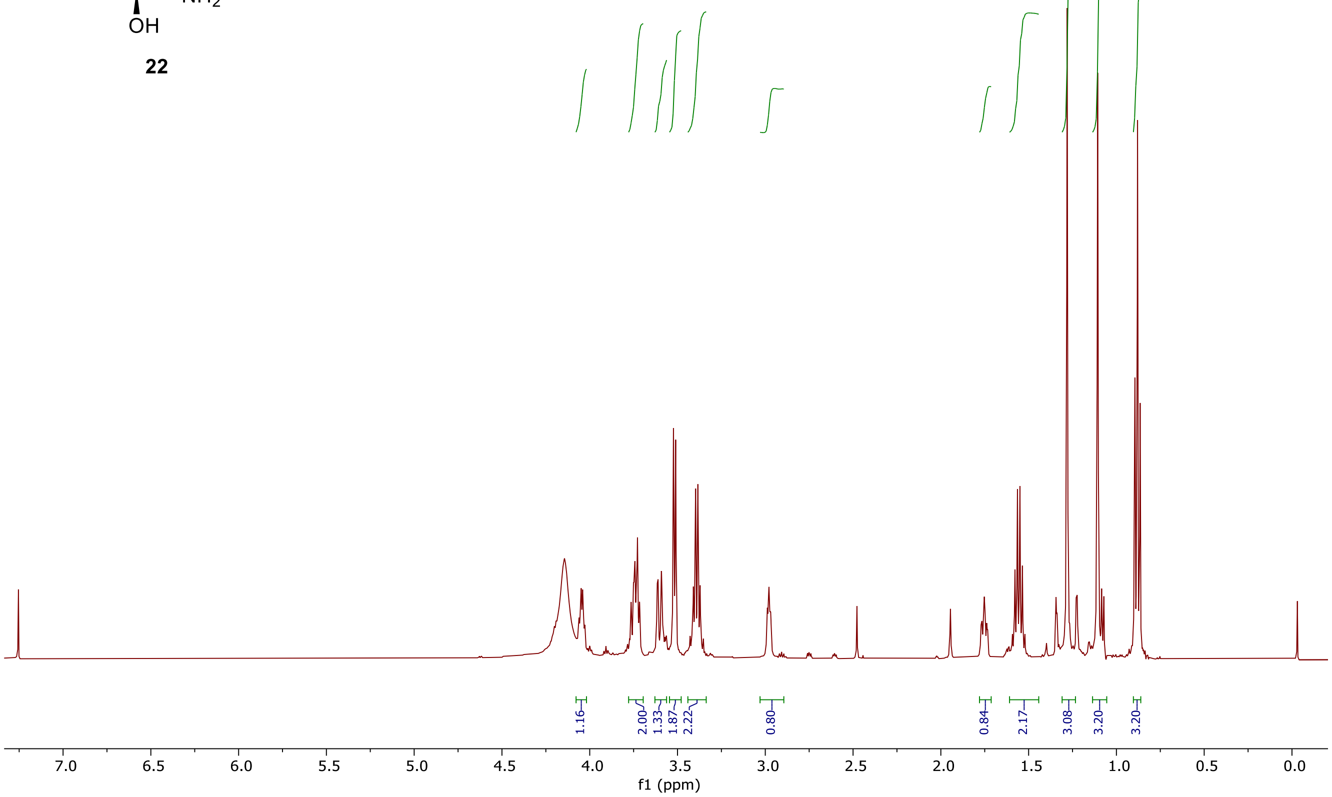
1.16
2.00
1.33
1.87
2.22
0.80
0.84
2.17
3.08
3.20
3.20
7.0 6.5 6.0 5.5 5.0 4.5 4.0 3.5 3.0 2.5 2.0 1.5 1.0 0.5 0.0
f1 (ppm)

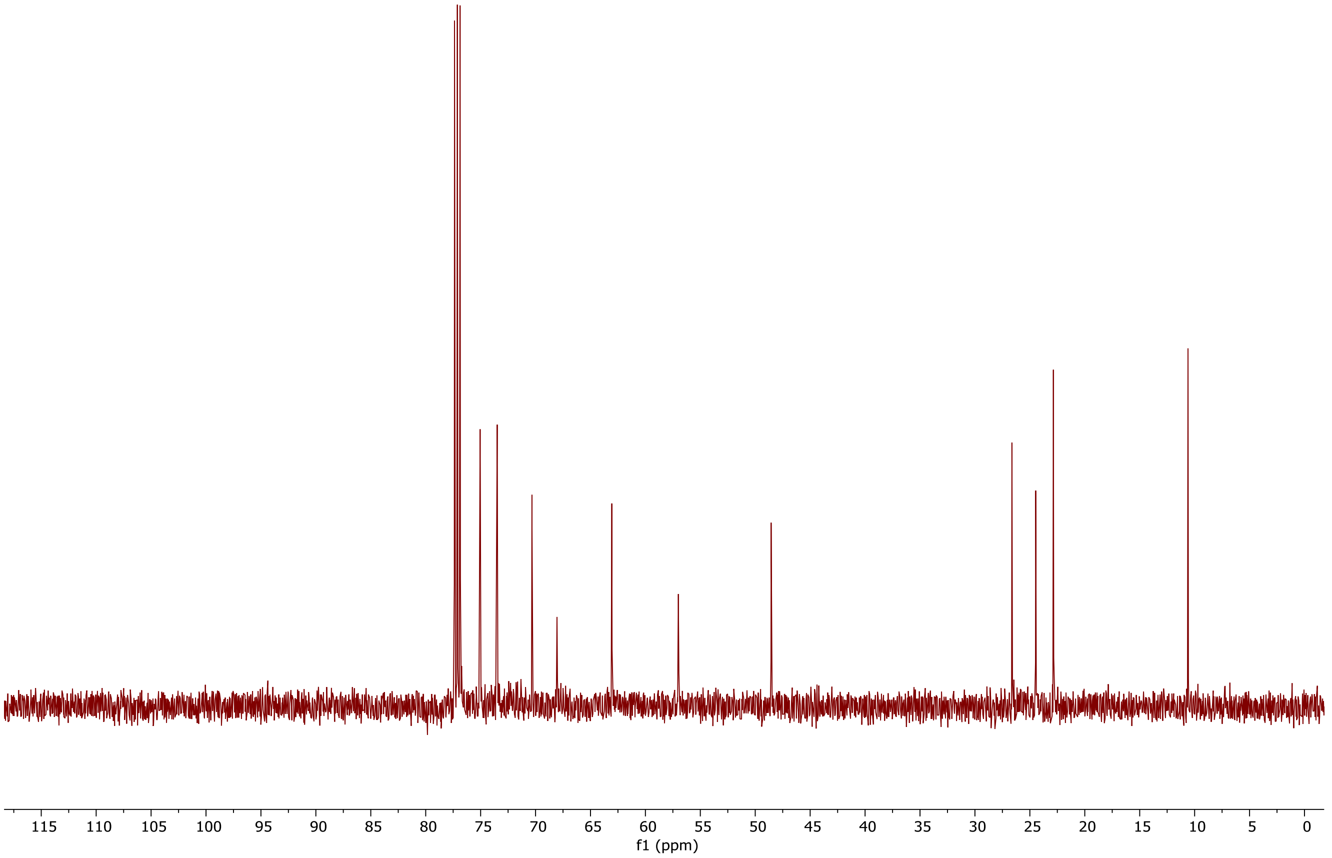
115 110 105 100 95 90 85 80 75 70 65 60 55 50 45 40 35 30 25 20 15 10 5 0
f1 (ppm)

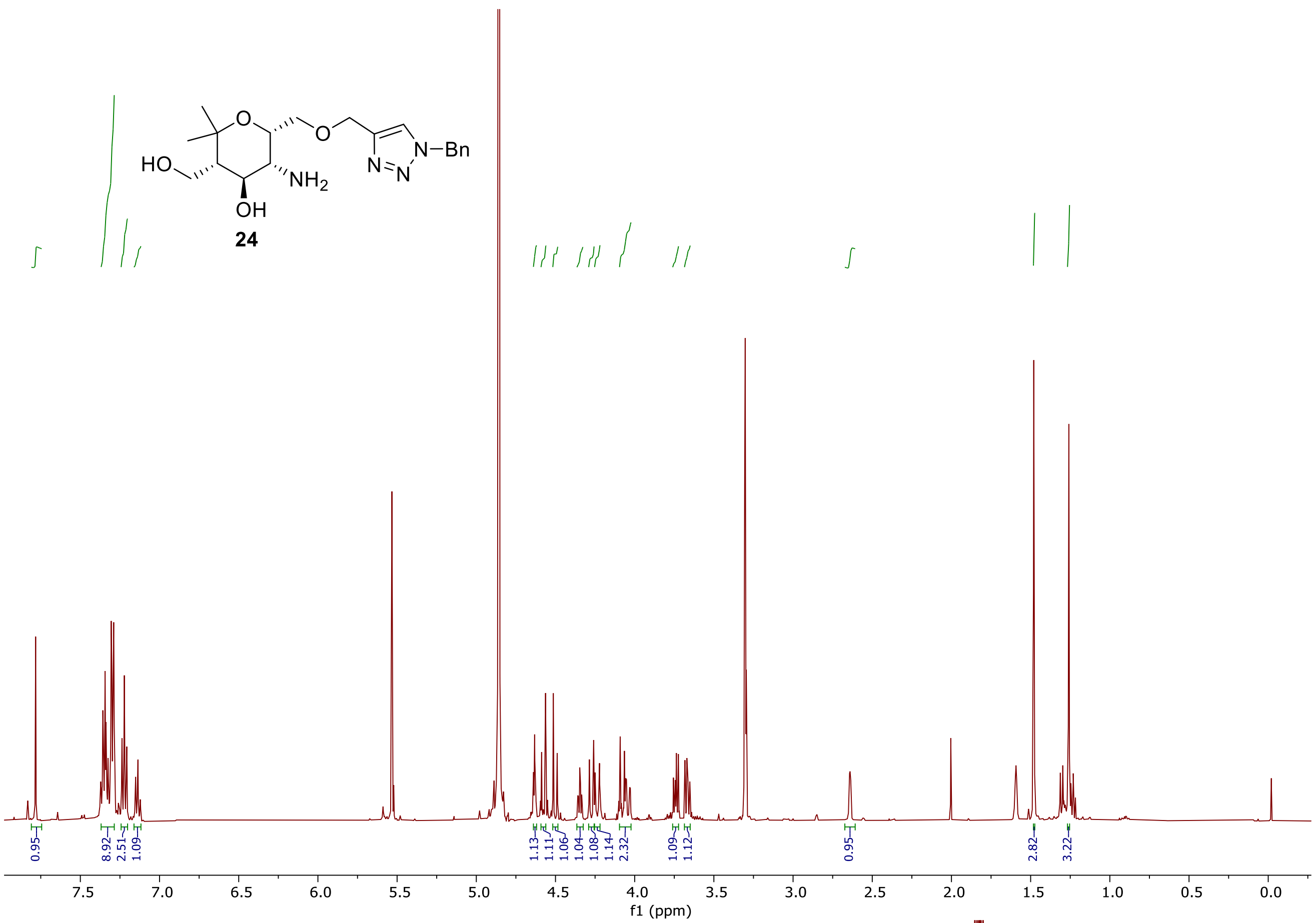
HO
O
NH2
OH
N
N
N–Bn
24
f1 (ppm)

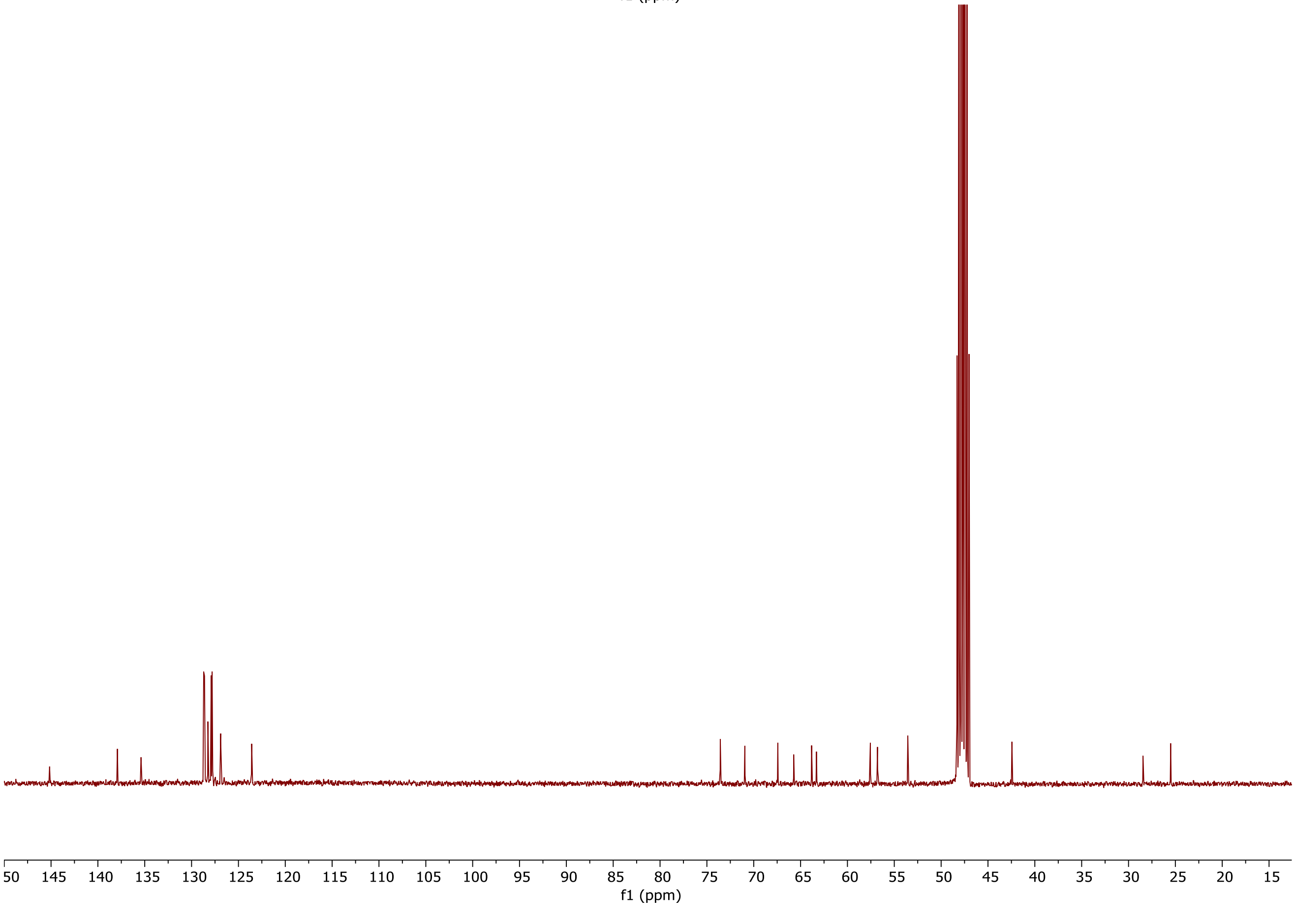
f1 (ppm)

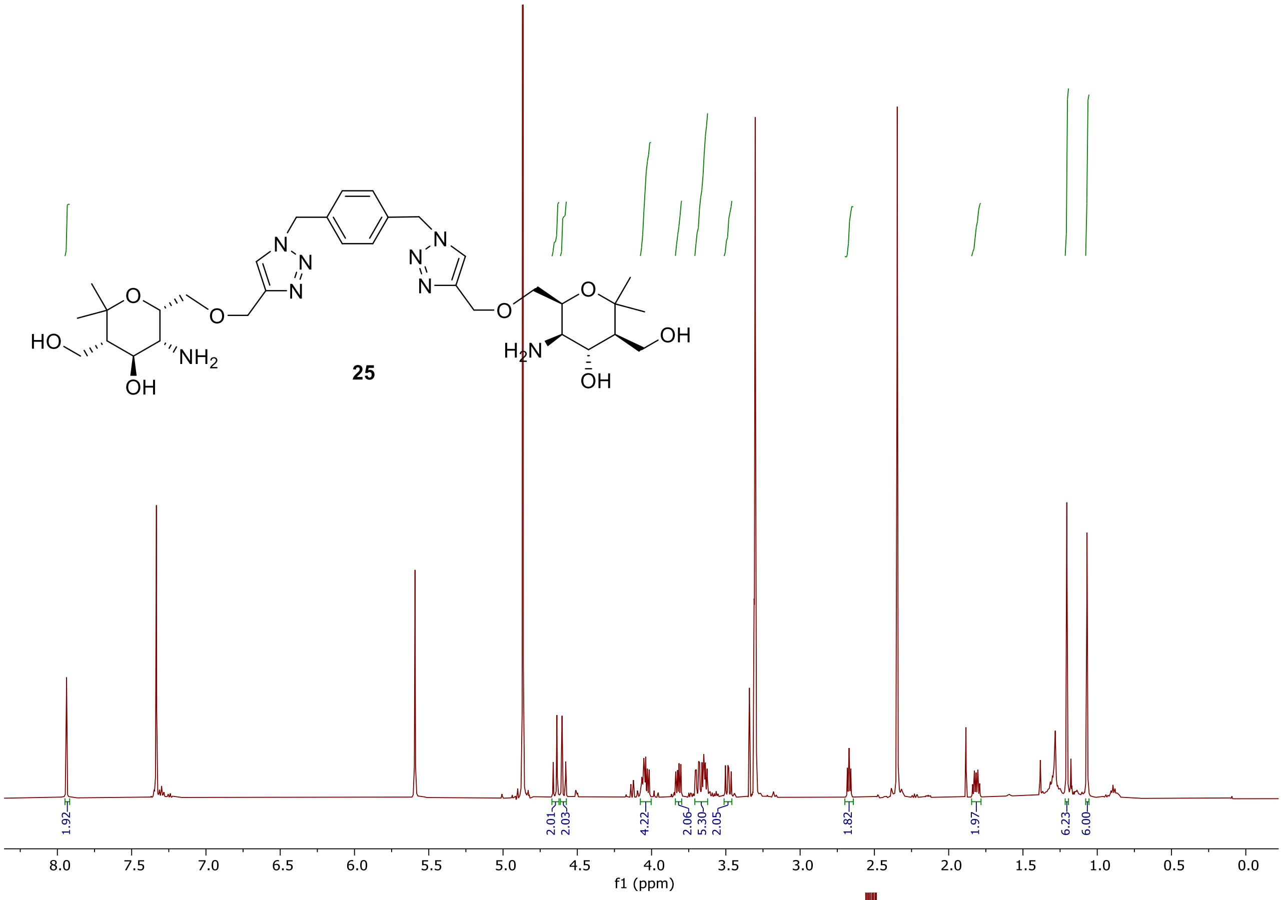
HO
OH
NH2
H2N
OH
OH
25
1.92
2.01
2.03
4.22
2.06
5.30
2.05
1.82
1.97
6.23
6.00
8.0 7.5 7.0 6.5 6.0 5.5 5.0 4.5 4.0 3.5 3.0 2.5 2.0 1.5 1.0 0.5 0.0
f1 (ppm)

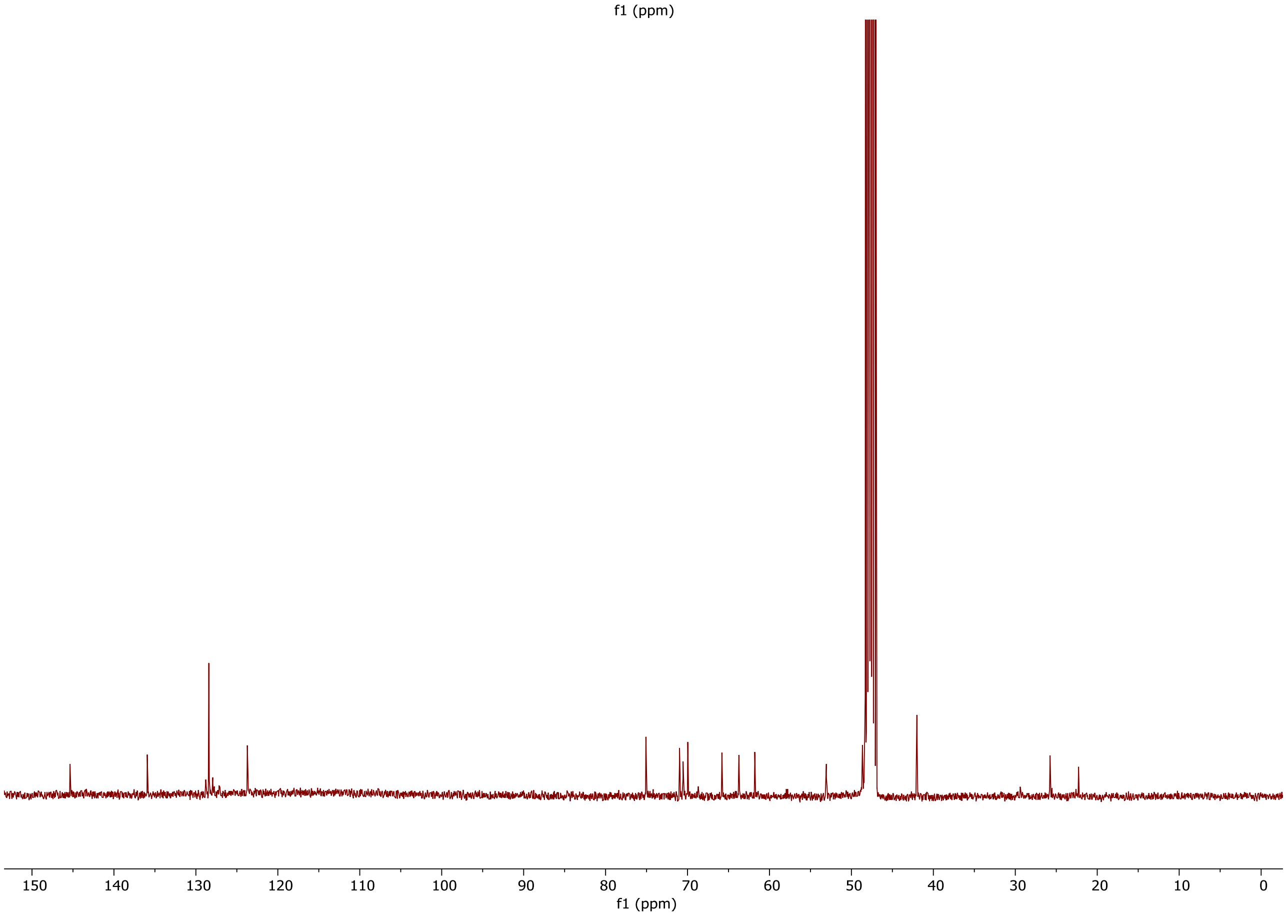
150 140 130 120 110 100 90 80 70 60 50 40 30 20 10 0
f1 (ppm)

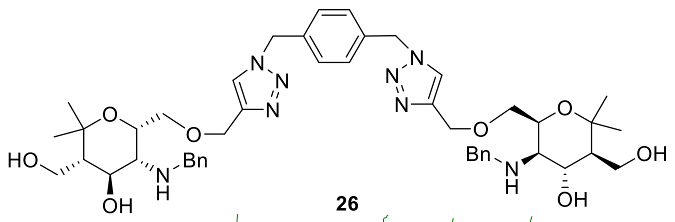
HO
OH
N
H
Bn
O
N
N
N
OH

26

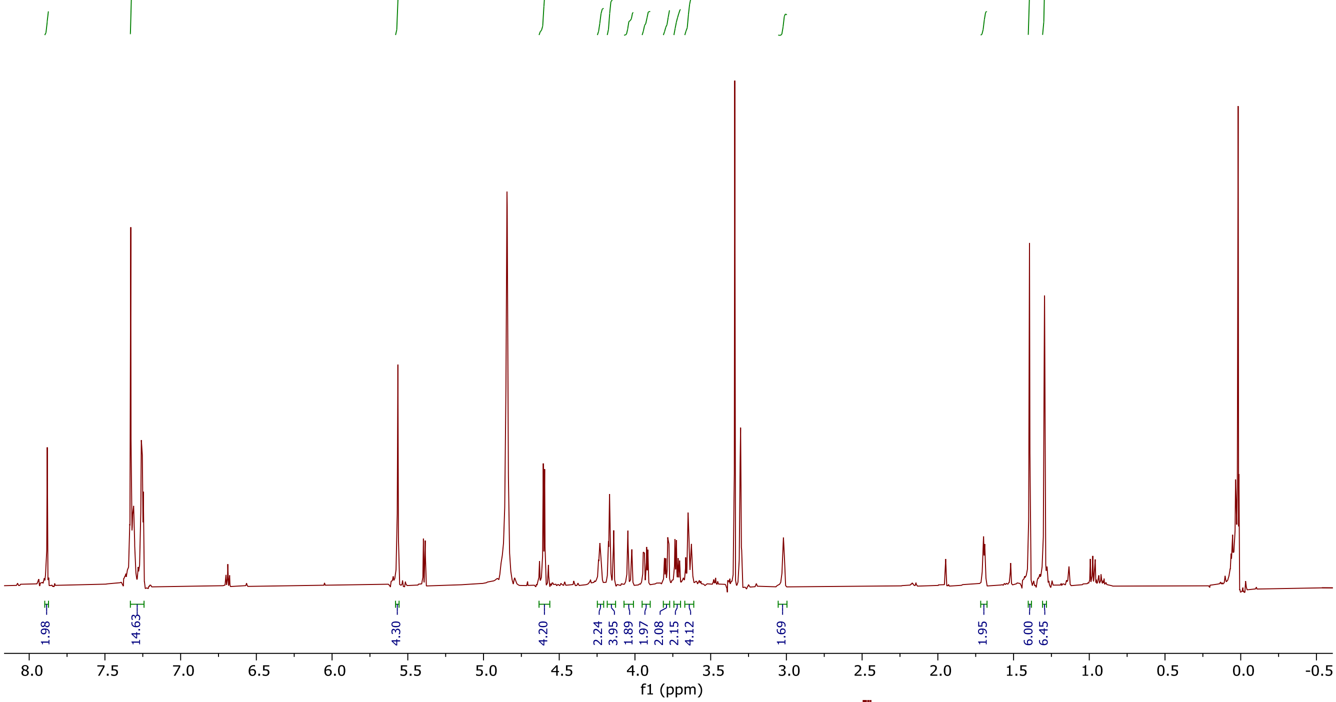
1.98
14.63
4.30
4.20
2.24
3.95
1.89
1.97
2.08
2.15
4.12
1.69
1.95
6.00
6.45
8.0 7.5 7.0 6.5 6.0 5.5 5.0 4.5 4.0 3.5 3.0 2.5 2.0 1.5 1.0 0.5 0.0 -0.5
f1 (ppm)

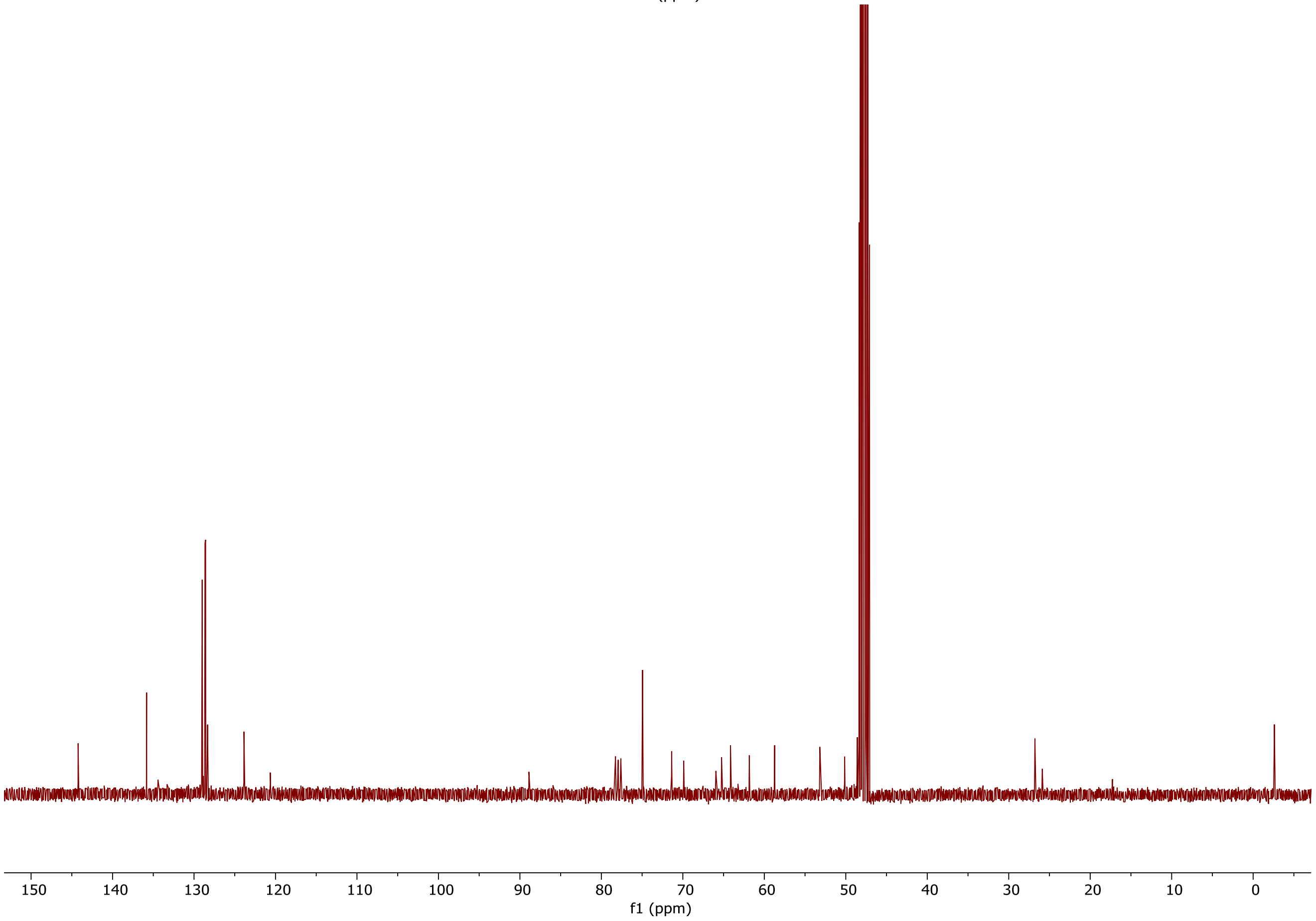
150 140 130 120 110 100 90 80 70 60 50 40 30 20 10 0
f1 (ppm)
